# Mediator subunit MED25 represses ABI5-dependent activation of erucic acid biosynthetic gene *FAE1* in *Brassica napus*

**DOI:** 10.3389/fpls.2026.1798651

**Published:** 2026-06-10

**Authors:** Zhuang Li, Shouchuan Zhi, Yongkang Tang, Huan Li, Mengliang Tian, Xiaobin Pu, Yongcheng Wu

**Affiliations:** 1 State Key Laboratory of Crop Gene Exploration and Utilization in Southwest China, Sichuan Agricultural University, Chengdu, China; 2 College of Agronomy, Sichuan Agricultural University, Chengdu, China; 3 Crop Research Institute of Sichuan Academy of Agricultural Sciences (Sichuan Germplasm Resources Center), Sichuan Academy of Agricultural Sciences, Chengdu, China

**Keywords:** ABI5, *Brassica napus*, erucic acid, FAE1, MED25

## Abstract

ABSCISIC ACID INSENSITIVE 5 (ABI5) is a key regulator that represses seed germination, flowering, and seedling growth in *Arabidopsis thaliana* and functions in abscisic acid (ABA) signal transduction. However, whether ABI5 is involved in other biological processes and its interacting partners remain largely unknown. Here, we show that *BnaABI5*, an ortholog of *AtABI5*, positively regulates erucic acid synthesis and the transcript level of the marker gene *FAE1* in *Brassica napus*. Expression patterns of *BnaABI5* and *BnaFAE1* overlap during specific stages of seed development, as indicated by the BnIR database, and GUS staining assays show that both genes are expressed in seeds. BnaABI5 is a bZIP transcription factor that localizes to the nucleus and activates transcription of the reporter gene *LacZ*. We further demonstrate that the mediator subunit BnaMED25 is involved in modulating erucic acid synthesis through interaction with BnaABI5 *in vivo* and *in vitro*. Dual-luciferase assays and electrophoretic mobility shift assays (EMSAs) indicate that BnaMED25 facilitates BnaABI5-mediated regulation of *BnaFAE1* expression. Genetic experiments indicate that, compared with wild-type Col-0 and the variety “Wangxiangyou1881”, the transcript level of the marker gene *FAE1* is increased in *BnaABI5*-overexpressing *Arabidopsis* and oilseed rape, respectively. Erucic acid content in the tested seeds exhibited a corresponding trend. Taken together, these results demonstrate that BnaMED25 is involved in BnaABI5-mediated regulation of erucic acid accumulation and reveal a mechanism by which ABI5 and its recruiting factor play a significant role in long-chain fatty acid synthesis.

## 1 Introduction

*Brassica napus* L., commonly known as oilseed rape, is a major oilseed crop that is widely cultivated worldwide (Song et al., 2020a). As an allotetraploid species (AACC, 2n = 38), *B. napus* originated from natural interspecific hybridization between two diploid species, *Brassica oleracea* (CC, 2n = 18) and *Brassica rapa* (AA, 2n = 20). Low erucic acid content is considered beneficial for human health and for edible oil production, whereas high erucic acid content has important industrial applications, including use in lubricants, plastics, and chemical manufacturing (Reyes et al., 1995). In recent years, biofuels have gained widespread acceptance due to their potential for environmental protection and the depletion of fossil fuel resources (Russo et al., 2025). Therefore, altering the fatty acid composition of *B. napus* seeds is a critical objective for genetic improvement of oilseed rape.

In general, triacylglycerol (TAG) biosynthesis is a complex regulatory network that comprises three main biological processes: glycolysis, *de novo* fatty acid synthesis, and TAG assembly (Yang et al., 2022). Multiple transcription factors have been shown to be involved in regulating oil biosynthetic pathways in plants. WRINKLED1 (WRI1), first identified in *Arabidopsis thaliana* (Focks and Benning, 1998), plays a crucial role in regulating the expression of numerous genes involved in glycolysis and fatty acid synthesis. For example, WRI1 regulates genes encoding plastidic enzymes, including pyruvate kinase, the pyruvate dehydrogenase E1α subunit, the acetyl-CoA carboxylase BCCP2 subunit, enoyl-ACP reductase, and phosphoglycerate mutase (Baud et al., 2007). Other transcriptional regulators participate in fatty acid synthesis by regulating *WRI1* expression. LEAFY COTYLEDON1 (LEC1) has been shown to bind to WRI1 using chromatin immunoprecipitation assays (Pelletier et al., 2017), and regulation of *WRI1* expression by LEAFY COTYLEDON2 (LEC2) has also been demonstrated through analysis of lipid biosynthesis-related gene expression in transgenic *Arabidopsis* (Kim et al., 2015). Furthermore, chromatin immunoprecipitation on chip (ChIP–chip) assays have identified *WRI1* as a target gene of FUS3 and ABI3 during lipid biosynthesis (Wang and Perry, 2013; Tian et al., 2020).

Over the past 15 years, significant progress has been made in understanding the roles of additional transcription factors in regulating oil biosynthesis. Numerous transcription factors have been shown to be involved in modulating oil biosynthesis. MYB96 participates in fatty acid synthesis by regulating the expression of *DGAT1* and *PDAT1* (Lee et al., 2018). MYB76 influences oil biosynthesis by downregulating two *GDSL-*like lipase genes (AT1G71691 and AT4G01130) and several genes associated with lipid metabolism, including *BIOTIN ATTACHMENT DOMAIN-CONTAINING 2* (*BADC2*), *GLNB1 HOMOLOG* (*GLB1*), *ACYL CARRIER PROTEIN 5* (*ACP5*), ACYL-ACP *DESATURASE 1* (*AAD1*), and *OLEOSIN 3* (*OLEO3*), while upregulating key genes involved in fatty acid synthesis, such as *SUCROSE SYNTHASE 4* (*SUS4*) and *3-KETOACYL-CoA SYNTHASE 17* (*KCS17*), in the *myb76–2* mutant at different days after pollination (DAP) (Duan et al., 2017). TRANSPARENT TESTA GLABRA1 (TTG1), a WD40 domain-containing protein, mediates fatty acid biosynthesis by indirectly inhibiting the expression of oil biosynthesis-related genes (Chen et al., 2015). WRKY43 participates in fatty acid desaturation, as evidenced by altered polyunsaturated fatty acid content in *wrky43* loss-of-function lines (Geilen et al., 2017). WRKY6 negatively regulates the expression of *FUS3* and *DGAT1*; consequently, *wrky6* mutants exhibit increased seed oil content (Song et al., 2020b). A soybean tandem CCCH zinc finger (TZF) protein, GmZF392, enhances oil accumulation when overexpressed in *Arabidopsis* by modulating the expression of oil biosynthesis genes (Lu et al., 2021). In addition to these regulators, extensive research has been conducted on other proteins that participate in regulating lipid biosynthesis. DGAT1 and DGAT2 are responsible for catalyzing the conversion of diacylglycerol (DAG) to TAG (Busta et al., 2022). Oil accumulation is enhanced in transgenic plants through the overexpression of *AtDGAT1* in *Jatropha curcas* and *CeDGAT2–2* in *Cyperus esculentus*, respectively (Maravi et al., 2016; Gao et al., 2021). Recently, homologs of *DGAT1* from multiple species have been expressed in a lipid-deficient yeast mutant. In this yeast expression system, *DGAT1* homologs from *Vernonia*, sunflower, *Jatropha*, and sesame resulted in significant increases in TAG content (Hatanaka et al., 2022). Oleosin is a plant-specific alkaline protein associated with oil bodies (Pyc et al., 2017). Increased fatty acid content in tobacco leaves has been attributed to the heterologous overexpression of *SiOLE1* (Yee et al., 2021). Experimental evidence demonstrates that targeted modifications of conserved domains and specific amino acid residues in oleosin (OLE) proteins significantly enhance their lipogenic capacity in both *A. thaliana* and *Sesamum indicum* (Winichayakul et al., 2013; Anaokar et al., 2024).

Abscisic acid (ABA) is a key phytohormone that regulates plant stress responses and developmental transitions. As a key member of the bZIP transcription factor family in ABA signaling, ABI5 regulates the transcription of downstream target genes involved in abiotic stress tolerance and developmental transitions (Lim et al., 2013; Han et al., 2012; Lopez-Molina et al., 2002; Brocard et al., 2002; Finkelstein et al., 2005). For instance, ABI5 activates downstream regulatory networks that control embryonic patterning during seed morphogenesis and orchestrates maturation processes, including desiccation tolerance and reserve compound accumulation associated with seed longevity (Cheng et al., 2014; Finkelstein and Lynch, 2000; Xu et al., 2022; Zinsmeister et al., 2016). Acting as a signaling integrator, ABI5 modulates ABA responses through multiple mechanisms, including direct protein–protein interactions and regulation of its own gene expression, thereby facilitating crosstalk between ABA and other hormonal pathways. For example, RGL2 inhibits seed germination by promoting ABA biosynthesis and enhancing ABI5 activity (Piskurewicz et al., 2008). ICE1 regulates the expression of *EM1* and *EM6* through direct physical interaction with ABI5. Furthermore, during seed germination, DELLA proteins regulate ABA signaling by stabilizing ABI5 and forming a physical complex with ICE1 (Hu et al., 2019). BIN2 regulates ABA-mediated signaling in germinating seeds by forming protein complexes with ABI5 and enhancing its post-translational stability (Hu and Yu, 2014). The circadian oscillator component PRR5 coordinates seed germination through direct protein–protein interaction with ABI5 and by upregulating its transcriptional activity (Yang et al., 2021). Beyond its role in seed morphogenesis, ABI5 also regulates diverse developmental processes across plant life stages. For example, ABI5 is involved in regulating apple leaf senescence by positively regulating the transcription of *NYE1* and *NYC1* (An et al., 2021). In legumes, *abi5* mutants exhibit compromised raffinose family oligosaccharide (RFO) biosynthesis, reduced accumulation of late embryogenesis abundant (LEA) proteins, and paradoxical upregulation of photosynthesis-associated nuclear gene (PhANG) expression (Zinsmeister et al., 2016). In *A. thaliana*, ABI5 coordinates seed maturation by forming protein complexes with FRI and directly activating FLC transcription (Xu et al., 2022).

The Mediator complex is a multi-subunit eukaryotic assembly that functions as a transcriptional co-activator through coordinated protein–protein interactions (Zhai and Li, 2019; Soutourina, 2018; Poss et al., 2013) and was initially isolated in *A. thaliana* (Bäckström et al., 2007). The Mediator complex comprises multiple evolutionarily conserved subunits, including MED2, MED5, MED14, MED15, MED16, MED18, MED21, MED23, and MED25, which collectively contribute to its modular organization and transcriptional regulatory functions. For instance, the *ref4–3* mutation in MED5b represses phenylpropanoid accumulation. Mutations in MED2, MED16, and MED23 are also involved in phenylpropanoid biosynthesis (Dolan et al., 2017). In *A. thaliana*, NRB4, the functional ortholog of MED15, regulates salicylic acid (SA)-mediated defense signaling in plant immunity (Canet et al., 2012). Mutation of MED16 in *A. thaliana* disrupts NPR1 protein homeostasis and increases susceptibility to both bacterial and fungal pathogens, highlighting its role in immune regulation (Zhang et al., 2012). MED25, a core subunit of the Mediator complex, has been characterized as a key regulator of flowering time in plants (Cerdán and Chory, 2003); subsequent studies have further demonstrated its multifaceted roles in diverse developmental processes. AtMED25 modulates jasmonic acid (JA) signaling homeostasis by recruiting PRP39a and PRP40a, thereby inducing alternative splicing of *JAZ* genes (Wu et al., 2020). AtMED25 is involved in JA and ABA signaling by interacting with MYC2 and ABI5, respectively (Chen et al., 2012a). In tomato, SlMED25 controls fruit ripening through its interaction with EIL3 (Deng et al., 2023). In rice, OsMED25 modulates plant architecture through interaction with OsBZR1 and controls spikelet number via DST (Ren et al., 2020; Lin et al., 2022). These findings indicate that MED25 is a key regulator of plant growth and development. However, the role of MED25 in fatty acid biosynthesis and its underlying mechanisms remain unclear.

Here, we investigate how MED25 regulates *FAE1* expression through its interaction with ABI5 during erucic acid biosynthesis. We found that the expression patterns of these genes exhibit tissue-specific overlap, particularly in seeds during post-fertilization development. We demonstrate that ABI5 enhances the transcription of genes involved in erucic acid biosynthesis. Furthermore, MED25 suppresses ABI5-mediated transcriptional activation of the erucic acid biosynthetic gene *FAE1* through direct interaction. In addition, genetic experiments reveal that ABI5 positively regulates erucic acid biosynthesis by enhancing *FAE1* expression in *A. thaliana* and oilseed rape. Collectively, these findings illustrate how a Mediator subunit integrates transcription factor inputs to regulate erucic acid biosynthesis.

## 2 Results

### 2.1 Characterization of the transcription factor BnaABI5

**Figure 1:**
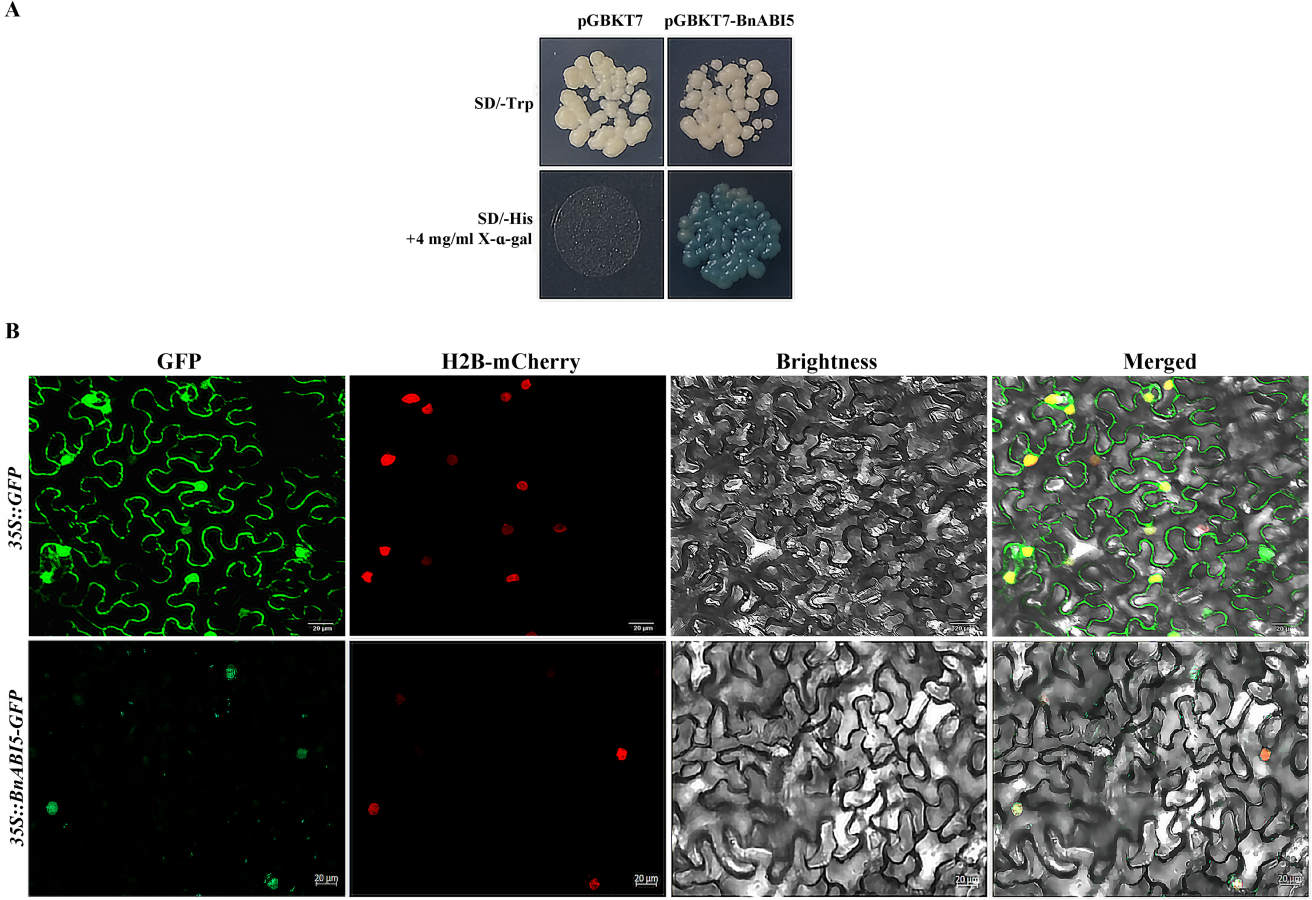
Characterization of BnaABI5. **(A)** BnaABI5 activates reporter gene transcription. (Top) Yeast cells transformed with empty pGBKT7 or pGBKT7–BnaABI5 were grown on synthetic defined (SD) medium lacking tryptophan (Trp). (Bottom) The same transformants were grown on SD medium lacking histidine (His) and supplemented with X-α-gal. **(B)** Subcellular localization of BnaABI5–GFP in *N. benthamiana* leaves. (Top) Empty GFP and H2B–mCherry were used as a control and a nuclear localization marker, respectively; (bottom) BnaABI5–GFP. Scale bars: 20 µm. All experiments were performed with three biological replicates and yielded consistent results.

Previous studies demonstrated that the AtABI5 protein contains a bZIP domain, indicating that *AtABI5* encodes a basic leucine zipper transcription factor belonging to the bZIP protein family (Finkelstein and Lynch, 2000). Homologs of *Arabidopsis* ABI5 have been identified in rice (Sakuraba et al., 2020), wheat (Ju et al., 2019), maize (Yan et al., 2012), and other species (Song et al., 2022b; Cui et al., 2021; Yeap et al., 2017), where they regulate the transcription of downstream genes across diverse biological processes. Transcription factors regulate the transcriptional levels of downstream target genes by acting as either transcriptional activators or repressors (Iwase et al., 2017; Wang et al., 2021a; Shang et al., 2010; Nakata et al., 2013). To determine whether BnaABI5 functions as a transcriptional activator or repressor, a transcriptional activation assay was performed in a yeast expression system. When the pGBKT7 empty vector or pGBKT7-BnaABI5 construct was transformed into the AH109 yeast strain, growth was observed on SD selective medium lacking tryptophan (Trp) (**Figure 1A**, top row). However, only yeast cells expressing pGBKT7-BnaABI5, and not the empty vector, were able to grow on selective medium lacking histidine (His) (**Figure 1A**, bottom row). To further confirm that *BnaABI5* functions as a transcription factor, a subcellular localization assay was performed in tobacco cells. The full-length coding sequence (CDS) of BnaABI5 was inserted into pCAMBIA1302-GFP to generate a fusion protein. Following transient expression in *Nicotiana benthamiana* leaf cells, fluorescence from the BnaABI5-GFP fusion protein co-localized with the nuclear marker H2B-mCherry (Burman et al., 2018) in the nucleus (**Figure 1B**, bottom row). In contrast, fluorescence from the empty GFP control was observed predominantly in the cytoplasm (**Figure 1B**, top row). Together, these results indicate that BnaABI5 functions as a nuclear-localized transcription factor with transcriptional activation activity.

### 2.2 Expression profile analysis of *BnaABI5* in different tissues

ABI5 is a core transcription factor in the ABA signaling pathway, and *FAE1* is a key gene controlling erucic acid biosynthesis. PoABI5 has been shown to activate *PoFAD3* transcription by directly binding to its promoter (Li et al., 2022). Moreover, the upregulation of key lipid biosynthesis genes in transgenic *Arabidopsis*, including *FAD2* and *FAD3*, results from heterologous overexpression of *PoABI5* (Li et al., 2025). These studies support the view that ABI5 functions as a direct upstream regulator of oil biosynthesis genes. To determine whether *BnaABI5* is involved in regulating erucic acid biosynthesis, we analyzed the expression profiles of several oil accumulation-related genes, including *BnaABI5* and *BnaFAE1*, using the BnIR database (Yang et al., 2023). The results showed that four copies each of the *BnaABI5* genes (*BnaC04G0548800ZS*, *BnaA05G0087100ZS*, and *BnaC04G0102500ZS*) (**Supplementary Figure 1**) and two copies each of the *BnaFAD2* genes (*BnaA05G0427800ZS* and *BnaC05G0480500ZS*) (**Supplementary Figure 2**), *BnaFAD3* genes (*BnaA04G0191900ZS* and *BnaC04G0496200ZS*) (**Supplementary Figure 3**), and *BnaFAE1* genes (*BnaA08G0134700ZS* and *BnaC03G0745900ZS*) exhibited similar expression patterns during 30–60 DAP (**Supplementary Figure 4**).

**Figure 2:**
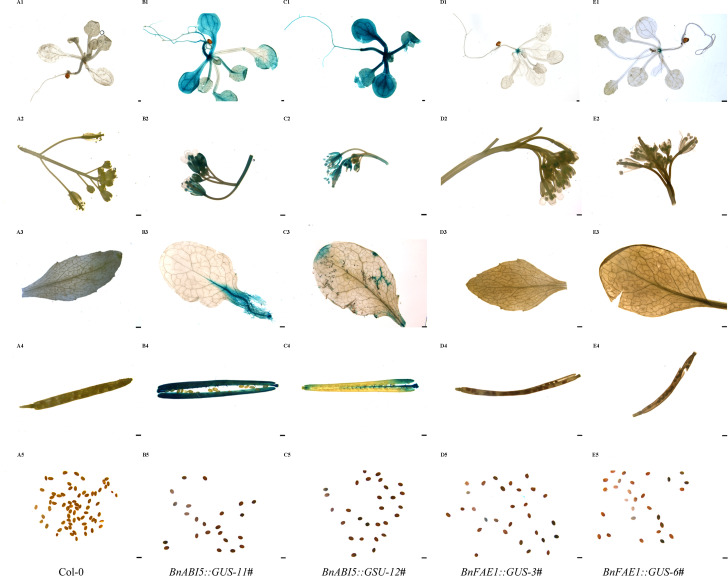
Expression patterns of *BnaABI5* and *BnaFAE1*. Corresponding tissues from non-transgenic wild-type Col-0 *Arabidopsis thaliana* were used as controls **(A)**. GUS staining of transgenic plants harboring 1,450 bp **(B, C)** and 1,310 bp **(D, E)** promoter fragments of *BnaABI5* and *BnaFAE1* fused to the GUS reporter gene. Seedlings, inflorescences, stems, leaves, siliques, and seeds (B1–B5 and C1–C5 for *BnaABI5*; D1–D5 and E1–E5 for *BnaFAE1*) were analyzed. Corresponding tissues from wild-type Col-0 *Arabidopsis thaliana* were used as controls. Scale bars: 500 µm. All experiments were performed with three biological replicates and yielded consistent results.

To further investigate whether BnaABI5 participates in regulating *BnaFAE1* expression, the promoter regions of *BnaABI5* and *BnaFAE1* were separately fused to the GUS reporter and introduced into wild-type *A. thaliana* (Col-0), and GUS staining was performed in the resulting transgenic lines. We first quantified GUS expression levels in different transgenic lines using semi-quantitative PCR and quantitative real-time PCR (qRT-PCR) (**Supplementary Figures 5**, **S6**). Histochemical staining was subsequently performed using a GUS staining kit. As expected, no GUS activity was observed in various tissues of wild-type Col-0 plants (**Figure 2**, A1–A5). Strong GUS activity was observed in multiple tissues of *BnaABI5_pro_::GUS-11*^#^ and *BnaABI5_pro_::GUS-12*^#^ lines, including whole seedlings, inflorescences, leaves, siliques, and seeds (**Figure 2**, B1–B5; **Figure 2**, C1–C5). In contrast, GUS activity in *BnaFAE1_pro_::GUS-3*^#^ and *BnaFAE1_pro_::GUS-6*^#^ lines was detected specifically in seeds, consistent with previous reports (Rossak et al., 2001; **Figure 2**, D1–D5; **Figure 2**, E1–E5). These expression patterns are consistent with the hypothesis that BnaABI5 may be involved in regulating *BnaFAE1*, although they do not establish a direct causal relationship. Subsequent experiments were conducted to verify this regulatory relationship. Collectively, these results suggest that *BnaFAE1* is a potential downstream target of BnaABI5 during oil accumulation.

### 2.3 *In vivo* and *in vitro* assays for BnaABI5 binding to the *BnaFAE1* promoter

**Figure 3:**
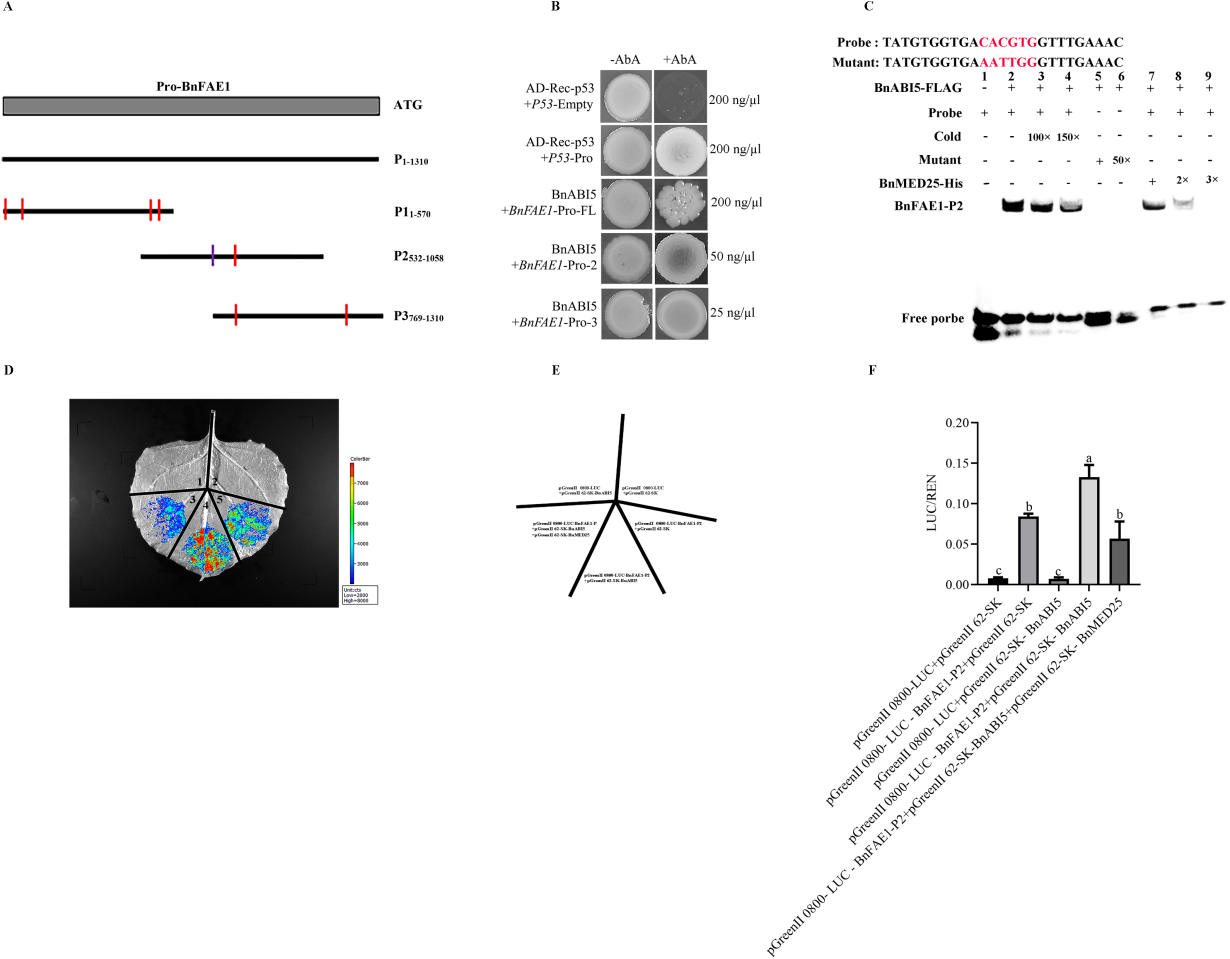
BnaMED25 inhibits BnaABI5-mediated transcriptional activation of *BnaFAE1.* **(A)** Schematic diagram of the full-length and truncated *BnaFAE1* promoter. Short vertical lines represent G-box or G-box-like motifs. LUC signals were analyzed in *N. benthamiana* leaves. **(B)** Yeast one-hybrid (Y1H) assays indicate that BnaABI5 regulates *BnaFAE1*. The empty pGADT7 vector and p53 promoter construct were used as negative and positive controls, respectively. **(C)** Electrophoretic mobility shift assay (EMSA) indicates that BnaABI5 directly binds to the *BnaFAE*1 promoter. Red-marked bases in the probe and mutated probe sequences indicate the G-box motif within the second truncated fragment of the *BnaFAE1* promoter and its corresponding mutated motif, respectively. **(D)** BnaABI5 regulates *BnaFAE1* transcription in *N. benthamiana* as assessed by dual-luciferase assays. Reporter fluorescence intensity reflects transcriptional activity. The BnaFAE1 promoter fragment was fused upstream of the LUC gene in the pGreenII 0800-LUC vector. Renilla luciferase (REN), driven by the CaMV 35S promoter in the pGreenII 0800-LUC vector, was used as an internal control. BnaABI5 and BnaMED25 were cloned downstream of the 35S promoter in the pGreenII 62-SK vector. The combination of empty pGreenII 62-SK and empty pGreenII 0800-LUC vectors served as the negative control. **(E)** Schematic illustration of the dual-luciferase assay. **(F)** Quantification of luciferase activity corresponding to **(D)**. Luciferase activity of the empty vector combination was normalized to 1. All experiments were performed with three biological replicates and yielded consistent results.

To investigate whether BnaABI5 binds to the *BnaFAE1* promoter, we performed a yeast one-hybrid (Y1H) assay. According to the BnIR database, two copies of the *BnaFAE1* gene, *BnaA08G0134700ZS* and *BnaC03G0745900ZS*, show significantly increased expression during seed development. We were unable to amplify the promoter sequence of *BnaA08G0134700ZS* by PCR. We also analyzed cis-regulatory motifs in the *BnaA08G0134700ZS* promoter using PlantCARE software. We identified only one ABRE-like motif in the *BnaA08G0134700ZS* promoter (data not shown), suggesting that BnaABI5 may bind to this promoter region. Therefore, the cloned promoter sequence of *BnaC03G0745900ZS* was selected for further analysis. Three truncated promoter fragments were generated such that each fragment contained one or two ABRE or ABRE-like cis-elements (**Figure 3A**; Jiang et al., 2024). Because the *BnaFAE1* promoter fragment spanning 1–570 bp (P1) exhibited self-activation that could not be suppressed by aureobasidin A (AbA) (Jiang et al., 2024), Y1H assays were performed using the full-length promoter and the remaining two fragments. As expected, no growth was observed in the negative control (**Figure 3B**, row 1). Similar to the positive control (**Figure 3B**, row 2), BnaABI5 bound to the full-length *BnaFAE1* promoter (**Figure 3B**, row 3). We next sought to determine which promoter fragment was responsible for binding. Y1H results indicated that the promoter fragments spanning 532–1,058 bp (P2) and 769–1,310 bp (P3), which contain ABRE or ABRE-like motifs, were bound by BnaABI5 (**Figure 3B**, rows 4, 5). To further validate this interaction, an electrophoretic mobility shift assay (EMSA) was performed. Because an ABRE motif was present in the second truncated promoter fragment (P2), this fragment was selected for subsequent EMSA analysis. Because purified recombinant BnaABI5 protein could not be obtained using a prokaryotic expression system (data not shown), the protein was instead produced using a cell-free expression system, and an EMSA was subsequently performed. The results showed that no shifted band was observed when only the probe was included (**Figure 3C**, lane 1). The recombinant BnaABI5-FLAG fusion protein exhibited strong binding to the ABRE motif in the *BnaFAE1* promoter (**Figure 3C**, lane 2). The addition of increasing amounts of unlabeled (cold) probe resulted in a progressive reduction in binding, indicating competitive inhibition (**Figure 3C**, lanes 3 and 4). In contrast, the recombinant protein showed no detectable binding to the mutated probe, even at high concentrations (**Figure 3C**, lanes 5 and 6). To further assess the regulatory relationship between BnaABI5 and *BnaFAE1 in vivo*, a dual-luciferase reporter assay was performed in *N. benthamiana*. The *LUC* reporter gene was placed under the control of the *BnaFAE1* promoter. One effector construct contained BnaABI5 driven by the CaMV *35S* promoter. A second effector construct was generated by cloning *BnaMED25* into the pGreenII 62-SK vector. A strong luminescence signal was observed when BnaABI5 and the *BnaFAE1* promoter fragment P2 were co-expressed in tobacco leaves (**Figure 3D**, injection point 4). In contrast, only weak signals were detected in the empty vector and negative controls (**Figure 3D**, injection points 1, 2, and 5). Taken together, these *in vitro* and *in vivo* assays demonstrate that BnaABI5 directly binds to the *BnaFAE1* promoter and positively regulates its expression.

### 2.4 BnaABI5 interacts with BnaMED25 *in vivo* and *in vitro*

**Figure 4:**
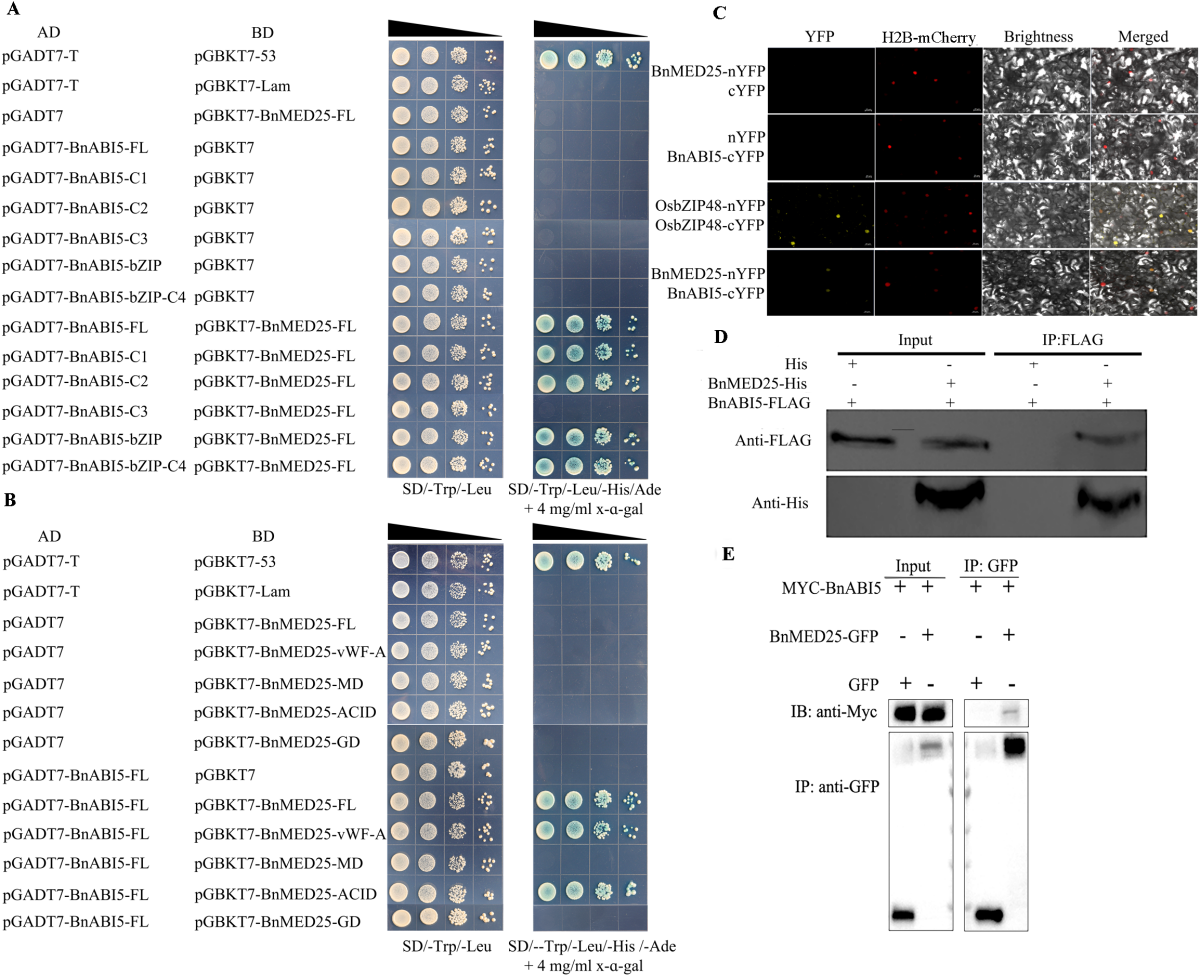
BnaABI5 physically interacts with BnaMED25. **(A, B)** Yeast two-hybrid (Y2H) assays showing interactions between full-length BnaMED25 and full-length BnaABI5, as well as their corresponding truncated domains in reciprocal configurations. Yeast transformants were grown on SD medium lacking leucine and tryptophan (SD/-Leu/-Trp) as a transformation control, or on SD medium lacking adenine, histidine, leucine, and tryptophan (SD/-Ade/-His/-Leu/-Trp) to assess protein–protein interactions. Co-transformations of empty pGBKT7 with empty pGADT7, empty pGBKT7 with BnaABI5 (panel A), and empty pGADT7 with BnaMED25 **(B)** were used as negative controls. **(C)** Bimolecular fluorescence complementation (BiFC) analysis showing that BnaABI5 interacts with BnaMED25. Images show confocal fluorescence micrographs of transiently transformed *N. benthamiana* epidermal cells co-expressing BnaMED25-nYFP and BnaABI5-cYFP. H2B-mCherry was used as a nuclear marker. Self-interacting OsbZIP48 served as a positive control, while co-expression of empty nYFP with BnaABI5 or empty cYFP with BnaMED25 served as negative controls. **(D)** Pull-down assays demonstrating the interaction between BnaABI5-His and BnaMED25-FLAG. BnaABI5-His and His alone were used as bait proteins, and BnaMED25-FLAG was used as the target protein. Bound proteins were analyzed by immunoblotting using anti-FLAG and anti-His antibodies. **(E)** Co-immunoprecipitation (Co-IP) assay showing the interaction between MYC-BnaABI5 and BnaMED25-GFP. Proteins extracted from *N. benthamiana* leaves transiently co-expressing MYC-BnaABI5 and BnaMED25-GFP were immunoprecipitated using anti-Myc magnetic beads, and interactions were detected using an anti-GFP antibody. All experiments were performed with three biological replicates and yielded consistent results.

AtMED25 is involved in seed germination and seedling development through its interaction with AtABI5. AtMED25 is subsequently recruited to the promoters of downstream genes, where it regulates their transcription (Chen et al., 2012a; You et al., 2019). MED25 has also been shown to be involved in regulating diverse biological processes in other species. In rice, MED25 interacts with DST to regulate spikelet number (Lin et al., 2022). Furthermore, OsMED25 has been shown to interact with OsBZR1 to regulate plant architecture in brassinosteroid signaling (Ren et al., 2020). We hypothesize that BnaMED25 is involved in erucic acid biosynthesis through its interaction with BnaABI5. To test this hypothesis, we performed a yeast two-hybrid (Y2H) assay using the Clontech yeast system. BnaABI5 was cloned into pGADT7 to generate the fusion construct pGADT7-BnaABI5, and BnaMED25 was cloned into pGBKT7 to generate pGBKT7-BnaMED25. To exclude the possibility that full-length BnaMED25 activates reporter gene transcription in yeast, a self-activation assay was conducted. We found that full-length BnaMED25 did not activate reporter gene transcription (**Figures 4A, B**, row 3). A Y2H assay was then performed to assess the interaction between BnaABI5 and BnaMED25. As expected, no interaction signal was observed in the negative control (**Figure 4A**, row 2). No interaction signals were observed in additional negative controls, including pGADT7 empty vector with full-length BnaMED25 and full-length BnaABI5 with pGBKT7 empty vector, under the same conditions (**Figure 4A**, rows 3, 9; **Figure 4B**, rows 3 and 8). Similar to the positive control (**Figure 4A**, row 1), the interaction between BnaABI5 and BnaMED25 was detected on selective medium lacking Trp, Leu, His, and Ade (**Figure 4A**, row 10). We also performed Y2H assays using reciprocal vector constructs. Similar interaction results were observed on selective media (**Figure 4B**, row 9). To identify the domains responsible for this interaction, we generated truncated derivatives of BnaABI5 and BnaMED25 and performed pairwise interaction assays. Under these conditions, none of the BnaMED25 derivatives exhibited self-activation of reporter gene transcription (**Figure 4B**, rows 4, 7). We found that three domains of BnaABI5, namely C1, C2, and the bZIP domain, are sufficient for interaction with BnaMED25 in yeast (**Figure 4A**, rows 11, 15). We also observed that two domains of BnaMED25, namely, the vWF-A and ACID domains, are sufficient for interaction with BnaABI5 (**Figure 4B**, rows 10, 13). The Y2H results were further validated using bimolecular fluorescence complementation (BiFC) assays. In BiFC assays, a strong yellow fluorescence signal was observed in the nucleus of tobacco cells co-expressing YC-BnaABI5 and YN-BnaMED25, similar to the positive control YC-OsbZIP48 and YN-OsbZIP48 (**Figure 4C**, row 3), and this signal co-localized with H2B-mCherry (**Figure 4C**, row 4; Burman et al., 2018). In contrast, no fluorescence signals were detected in negative controls, including YC with YN-BnaMED25 and YC-BnaABI5 with YN (**Figure 4C**, rows 1, 2). To further confirm the interaction between BnaMED25 and BnaABI5, *in vitro* pull-down assays were performed using purified fusion proteins. Because purified BnaMED25 and BnaABI5 proteins could not be obtained using a prokaryotic expression system, the pull-down assay was conducted using a cell-free expression system. The results showed that BnaABI5-FLAG was precipitated by BnaMED25-His, whereas His alone failed to pull down BnaABI5 (**Figure 4D**). In addition, co-immunoprecipitation (Co-IP) assays demonstrated that BnaABI5 and BnaMED25 exist in the same protein complex (**Figure 4E**). Collectively, these data demonstrate that BnaABI5 interacts with BnaMED25 both *in vivo* and *in vitro*.

### 2.5 BnaMED25 inhibits BnaABI5-mediated transcriptional activation of *BnaFAE1*

Previous studies have shown that MED25 negatively regulates ABA signaling by physically interacting with ABI5 during seed germination in *Arabidopsis* (Chen et al., 2012a). Additionally, MED25 in tomato is involved in fruit ripening through its interaction with EIL3 (Deng et al., 2023). Given that BnaMED25 interacts with BnaABI5, we investigated whether MED25 affects ABI5-mediated transcriptional regulation of *FAE1* during erucic acid biosynthesis in oilseed rape. To test this hypothesis, we performed a competitive EMSA. A reduction in BnaABI5-DNA binding was observed upon the addition of BnaMED25-His (**Figure 3C**, lane 7), and this inhibitory effect increased with higher concentrations of BnaMED25-His (**Figure 3C**, lanes 8, 9). To further validate the EMSA results, a dual-luciferase (LUC) reporter assay was performed in *N. benthamiana* leaves. Co-expression of BnaMED25 with BnaABI5 significantly attenuated BnaABI5-induced LUC expression (**Figure 3D**, injection point 3) compared with expression driven by BnaABI5 alone (**Figure 3D**, injection point 4). Collectively, these results suggest that BnaMED25 negatively regulates BnaABI5-mediated transcriptional activation of *BnaFAE1*.

### 2.6 Genetic evidence that BnaABI5 positively regulates erucic acid biosynthesis

**Figure 5:**
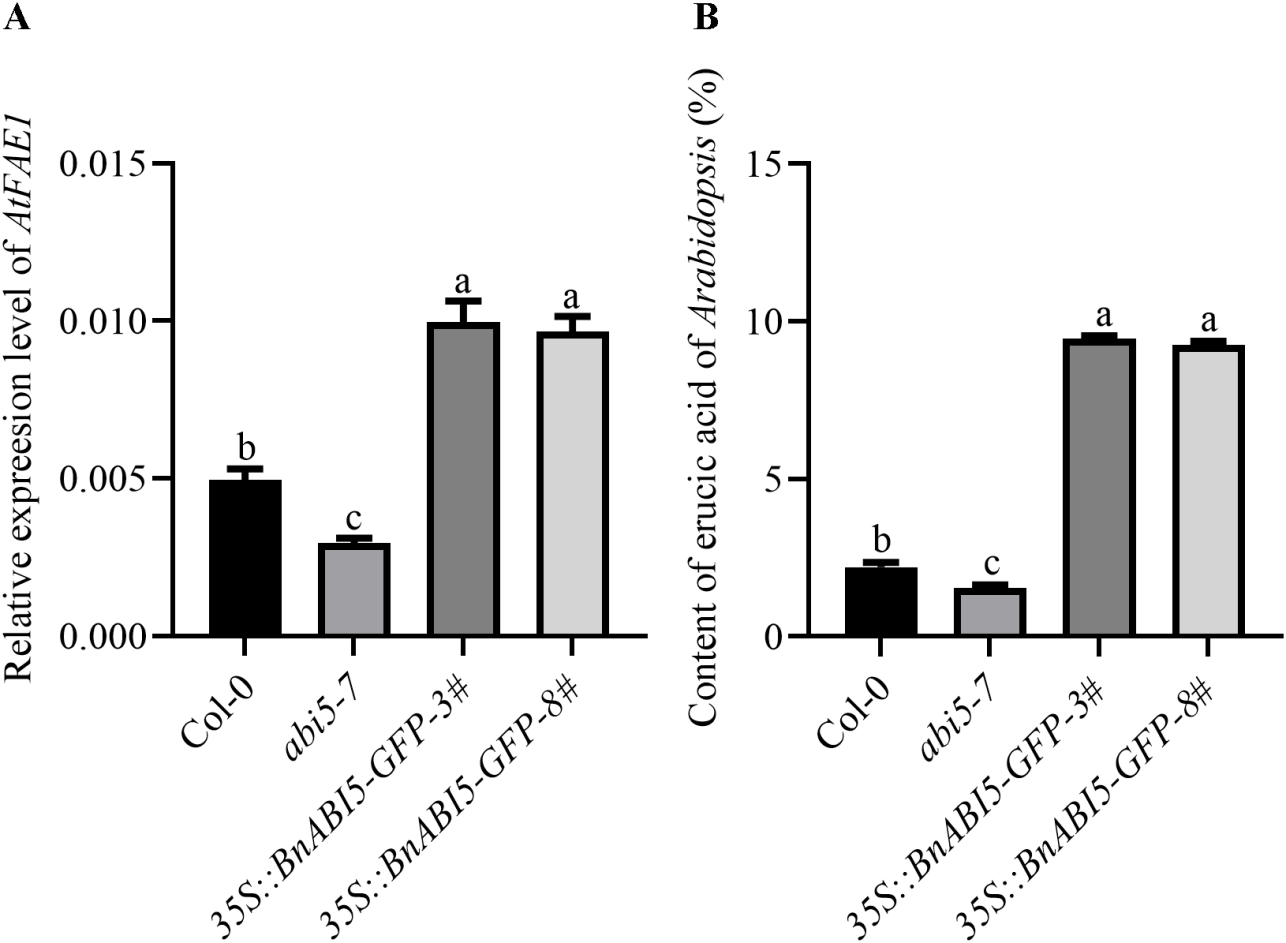
*BnaABI5* enhances erucic acid accumulation in *Arabidopsis*. **(A)** Relative expression levels of *AtFAE1* in wild-type (Col-0), *abi5–7* mutants, and transgenic *Arabidopsis* lines. *Actin2* was used as the internal reference gene. **(B)** Erucic acid content in *Arabidopsis* lines with different genetic backgrounds. Asterisks indicate statistically significant differences. Error bars represent the mean ± standard deviation (SD) of three biological replicates.

**Figure 6:**
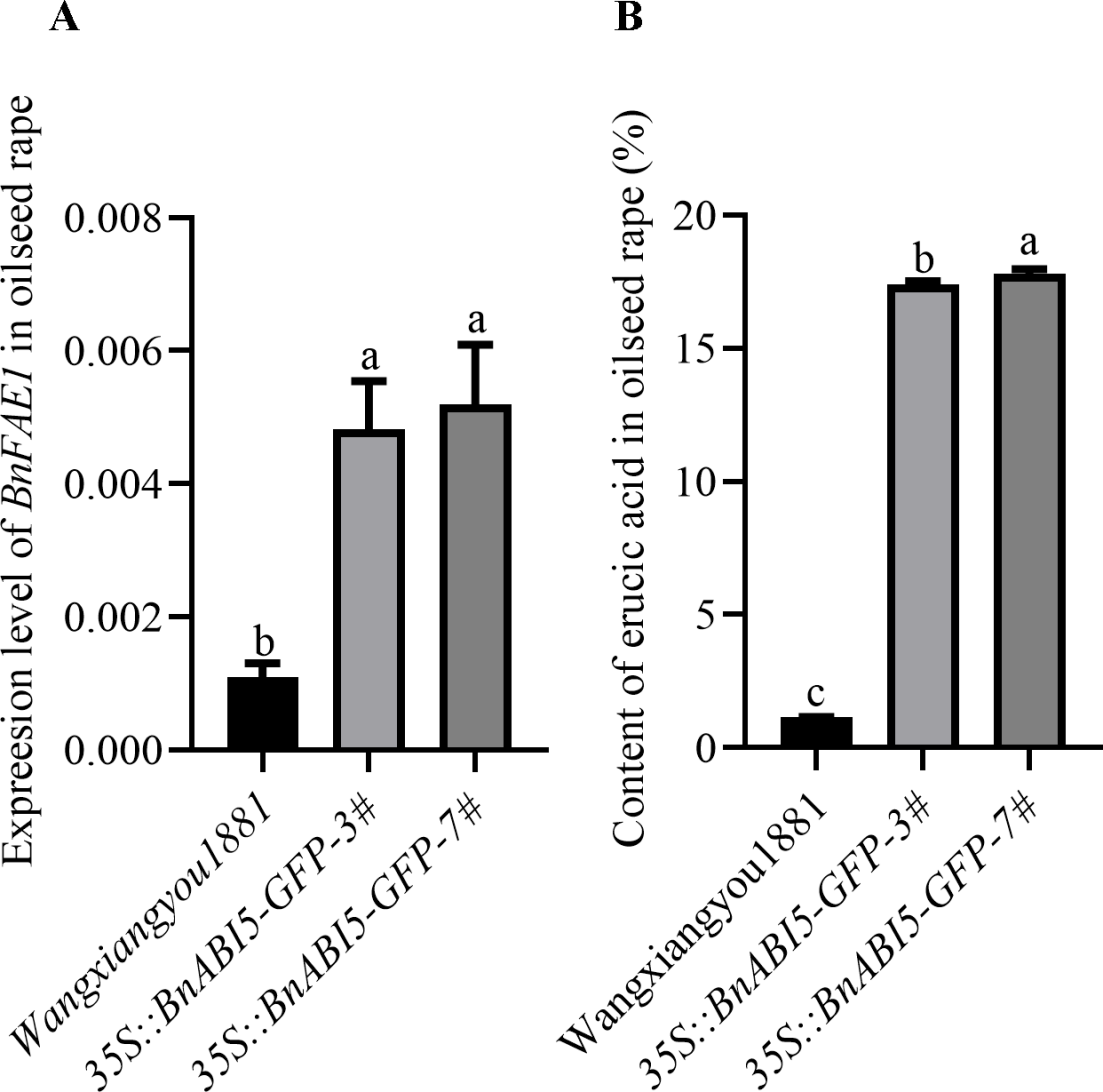
*BnaABI5* promotes erucic acid accumulation in oilseed rape (*Brassica napus*). Erucic acid content was measured using near-infrared spectroscopy in the cultivar “Wangxiangyou1881” and *BnaABI5*-overexpressing oilseed rape lines. **(A)** Relative expression levels of *BnaFAE1* in wild-type “Wangxiangyou1881” and transgenic oilseed rape lines. *Actin7* was used as the internal reference gene. **(B)** Erucic acid content in oilseed rape lines with different genetic backgrounds. Asterisks indicate statistically significant differences. Error bars represent the mean ± standard deviation (SD) of three biological replicates.

Molecular and biochemical analyses indicated that BnaABI5 positively regulates *BnaFAE1* expression, whereas BnaMED25 attenuates this effect (**Figure 3**). To further evaluate this regulatory role, we generated transgenic *Arabidopsis* (Col-0) lines overexpressing BnaABI5 and obtained homozygous lines. Expression of *BnaABI5* in transgenic lines was confirmed by semi-quantitative PCR and qRT-PCR (**Supplementary Figure 7**). The *Arabidopsis abi5–7* mutant was verified by sequencing (**Supplementary Figure 8**). The expression level of *AtFAE1* was then analyzed across different genetic background plants. *AtFAE1* expression was highest in overexpression lines 3 and 8 and lowest in the *abi5–7* mutant (**Figure 5A**). Erucic acid content in these lines showed trends consistent with *AtFAE1* expression levels (**Figure 5B**). In addition, *BnaABI5* was introduced into the oilseed rape variety “Wangxiangyou1881” and transgenic T2 plants were obtained. Expression of *BnaABI5* in transgenic plants was confirmed by semi-quantitative PCR and qRT-PCR (**Supplementary Figure 9**). The expression level of *BnaFAE1* was subsequently analyzed in different genetic background plants. *BnaFAE1* expression was significantly higher in transgenic lines than in wild-type “Wangxiangyou1881” (**Figure 6A**). Erucic acid content in these plants showed trends consistent with *BnaFAE1* expression levels (**Figure 6B**). Taken together, these genetic data support the conclusion that BnaABI5 is involved in erucic acid biosynthesis by positively regulating *BnaFAE1* expression.

## 3 Discussion

### 3.1 Hormone signaling regulators involved in fatty acid biosynthesis in plants

The synthesis and metabolism of fatty acids are key determinants of final oil quality. Previous studies have shown that, compared with the wild type, approximately 35% of genes are downregulated in the *wri1* mutant, many of which are involved in glycolysis and fatty acid metabolism, suggesting that WRI1 regulates oil accumulation by coordinating these pathways (Ruuska et al., 2002). Furthermore, overexpression of *AtWEI_1_* in soybean alters fatty acid composition and increases total fatty acid content (Vogel et al., 2019). In addition, the *EgWRI1–1* promoter in oil palm contains two CTTAAT motifs, two W-box elements, and one ABRE element, and its transcription is activated by EgNF-YA3, EgNF-YC2, and EgABI5 (Yeap et al., 2017). DELLA proteins, which are negative regulators of the gibberellin signaling pathway, play crucial roles in multiple aspects of plant development. Compared with the wild type, fatty acid composition is altered in gain-of-function mutants (bnaa6.*rga-D* and *ds-3*) of the DELLA family member BnaRGA. For example, the proportion of oleic acid decreases, whereas that of linoleic acid increases in these mutants (Yan et al., 2021). Recently, four genes—*BnaA09.PYRD*, *BnaA08.PSK1*, *BnaA08.SWI3*, and *BnaC02.LTP15—*have been identified through genome-wide association and transcriptome analyses as positive regulators of oleic acid content and negative regulators of erucic acid content (Zhang et al., 2025a). ABF2, a member of the ABF transcription factor family in the ABA signaling pathway, regulates the expression of genes involved in fatty acid biosynthesis, such as MdKASIII (Jing et al., 2025). Nevertheless, the factors involved in regulating erucic acid biosynthesis remain largely elusive. Specifically, key regulators within hormone signaling pathways that mediate erucic acid biosynthesis remain poorly understood. In this study, we isolated ABI5, a key regulator of the ABA signaling pathway, through PCR-based cloning. We demonstrate that the Mediator subunit BnaMED25 physically interacts with BnaABI5, thereby influencing erucic acid biosynthesis in oilseed rape.

### 3.2 Multiple factors interacting with ABI5 influence fatty acid biosynthesis

ABI5, a key regulator in the ABA signaling pathway, modulates multiple aspects of plant development. In apples, MdABI5 has been identified as an upstream activator of *MdMYBS1*, an R1-type MYB transcription factor whose transcriptional activity is strongly associated with carotenoid and ABA levels in fruits (Jia et al., 2024). In addition to regulating target gene transcription within the ABA signaling pathway, ABI5 also plays important roles in other hormone signaling pathways. MdAX2, a key regulator of the strigolactone signaling pathway, is involved in the induction of anthocyanin biosynthesis. MdABI5 directly binds to the MdAX2 promoter to activate its transcription, thereby enhancing anthocyanin accumulation in apple (Zhang et al., 2025b). In *Arabidopsis*, AtABI5 positively regulates *AtKRP1* transcription by directly binding to its promoter, which promotes the interaction between AtKRP1 and AtSTM. Thus, plants achieve a balance between coping with adverse stress conditions and maintaining growth and survival under drought conditions (Wang et al., 2024). MaABI5-like directly enhances cold tolerance in “Fenjiao” banana fruit and tomato, as transient overexpression in banana and heterologous overexpression in tomato increase cold resistance, whereas gene silencing produces the opposite phenotype (Song et al., 2022a). This effect is associated with the positive regulation of five fatty acid desaturation-related genes—*MaFAD3-1*, *MaFAD3-4*, *MaFAD3-5*, *MaFAD6-2*, and *MaFAD6-3*—by MaABI5-like. Conversely, silencing of MaABI5-like leads to reduced expression of these genes and decreased cold tolerance in both banana fruit and tomato (Song et al., 2022a). Furthermore, exogenous ABA treatment enhances linoleic acid accumulation by activating ABA signaling-related genes, including *ABI5*, which in turn upregulates *FAD2* expression (Shi et al., 2021; Kim and Li, 2022). In addition to ABI5, other regulators also participate in the modulation of fatty acid biosynthesis. In oil palm, EgMEDS16 negatively regulates the transcription of fatty acid-related genes, including *FAD2*, *SAD*, and *DGAT2*, during mesocarp development and interacts with EgGLO1 to influence fatty acid and TAG biosynthesis (Wang et al., 2021b). In *Arabidopsis*, AtbHLH7 positively regulates the synthesis of oleic and linoleic acids, while negatively regulating the synthesis of stearic, linolenic, and arachidic acids. The transcription factor AtPDF2, which interacts with AtbHLH7, positively regulates the synthesis of linoleic and linolenic acids while negatively regulating the synthesis of stearic, arachidic, and erucic acids. Moreover, AtbHLH7 and AtPEF2 act synergistically to inhibit seed oil accumulation by directly repressing fatty acid biosynthesis-related genes, including *MYB96*, *PDH-E1β1*, *PDH-E1β2*, and *BCCP1* (Liu et al., 2025). The APETALA2/Ethylene-Responsive Factor (AP2/ERF) transcription factor ERF13 inhibits the expression of 3-ketoacyl-CoA-synthase 16 (KCS16), which encodes a fatty acid elongase involved in very-long-chain fatty acid (VLCFA) biosynthesis (Lv et al., 2021). In addition, BAHD acyltransferases such as CER2 and CER2-like proteins function as components of the VLCFA elongation machinery by associating with specific KCS enzymes (Haslam et al., 2012) and are required for VLCFA biosynthesis (Haslam et al., 2015). These findings, together with our results demonstrating that BnaABI5 regulates erucic acid biosynthesis by modulating *BnaFAE1* expression (**Figures 3**, **5**, **6**), indicate that multiple factors, including BnaABI5, are involved in regulating fatty acid biosynthesis, forming a complex regulatory network.

### 3.3 BnaMED25 regulates *BnaFAE1* transcription through interaction with BnaABI5

The Mediator complex plays a crucial role in regulating diverse aspects of plant development. MED25, an important subunit of the Mediator complex, was originally characterized as a key regulator of flowering time (Cerdán and Chory, 2003). To date, Mediator subunits are well known to be recruited to the promoters of target genes through interactions with specific transcription factors (Davis et al., 2002; Bernecky et al., 2011). Currently, two mutually exclusive models have been proposed to explain the function of MED25: one posits that MED25 acts as a conventional transcriptional coactivator whose activity is lost upon physical isolation, whereas the other suggests that MED25 is specifically recruited to chromatin, where it actively recruits inhibitory complexes to mediate transcriptional repression. The data from this study support the second model: first, the MED25 T-DNA mutant, *pft1-1*, exhibited a markedly increased expression level of *AtFAE1*, distinct from that observed in the *abi5–7* mutant (**Supplementary Figure 10**); second, BnaMED25 was shown to directly interact with BnaABI5 in both *in vitro* and *in vivo* assays (**Figure 4**); third, dual-luciferase assays demonstrated that BnaMED25 significantly inhibits BnaABI5-mediated activation of *BnaFAE1* transcription (**Figure 3**). Collectively, these results support a model in which MED25 is specifically recruited to distinct chromatin loci, where it functions as a molecular scaffold to recruit transcriptional inhibitory complexes, thereby repressing target gene expression.

Previous studies have demonstrated that MED25 plays diverse roles in regulating multiple aspects of plant development. For instance, MED25 is recruited to the promoters of MYC2 and ABI5 target genes through physical interactions with MYC2 and ABI5, exerting a positive effect on MYC2-regulated transcription and a negative effect on ABI5-regulated transcription, respectively (Chen et al., 2012a). SlMED25 interacts with EIN3-like transcription factors and thus plays an essential role in fruit ripening (Deng et al., 2023). Our study further indicates that BnaMED25 plays a crucial role in erucic acid biosynthesis through interaction with BnaABI5. Nevertheless, differences in interaction domains are observed among species. In tomato, the ACID domain of SlMED25, rather than the full-length protein, is sufficient for interaction with SlABI5 in Y2H assays (Deng et al., 2023). A similar observation has been reported in *Arabidopsis* (Chen et al., 2012a). In addition, an amino acid fragment of AtABI5 (residues 221–349), rather than the full-length protein, is sufficient for interaction with AtMED25 in Y2H assays (Chen et al., 2012a). Somewhat unexpectedly, we found that multiple domains of BnaABI5, including the C1, C2, and bZIP domains, interact with BnaMED25 (**Figure 4A**). Similarly, the vWF-A and ACID domains of BnaMED25 mediate interaction with BnaABI5 (**Figure 4B**). In general, gene expression patterns and functions are often conserved across species. Variation in the expression patterns of homologous genes among species may result from differences in cis-regulatory elements within their promoter regions (Ma et al., 2013). Moreover, functional diversification may have occurred prior to monocot–dicot divergence, followed by the acquisition of additional functions during evolutionary history (Wu et al., 2019). We speculate that the expanded interaction domains observed for BnaMED25 reflect its capacity to integrate multiple signaling pathways through domain-specific protein interactions. Furthermore, these additional interaction regions suggest that, while conserving core functions shared with homologs in other species, BnaMED25 may have acquired novel regulatory functions during evolution.

### 3.4 Complex effects of erucic acid on plant growth and development

It is widely accepted that high levels of erucic acid and glucosinolates are harmful to human health, leading to the large-scale development of “double-low” (low erucic acid and low glucosinolate) rapeseed cultivars, while high-erucic-acid varieties are primarily utilized for industrial applications and derivative products. Erucic acid also plays roles in plant growth and development. Variation in seed fatty acid composition is crucial for plant responses to environmental stress, particularly erucic acid, a very-long-chain monounsaturated fatty acid, which significantly affects rapeseed germination under stress conditions (Wang et al., 2008). A recent study of 114 rapeseed germplasm accessions showed that, compared with low-erucic-acid seeds, high-erucic-acid seeds exhibit enhanced osmotic adjustment, enzyme activity, and hormonal regulation, resulting in higher germination rates and shorter mean germination times under drought stress conditions. Pretreatment of low-erucic-acid rapeseed seeds with different concentrations of erucic acid results in responses similar to those observed in high-erucic-acid seeds under the same stress conditions (Ai et al., 2024). Furthermore, high-erucic-acid rapeseed exhibits greater salt tolerance than low-erucic-acid rapeseed (Wang et al., 2008). However, erucic acid may exert opposite effects in other plant species. For instance, in wheat, erucic acid concentration is negatively correlated with malt fresh weight and positively correlated with peroxidase activity in buds and roots, indicating that erucic acid functions as an allelopathic inhibitory compound (Yue et al., 2018). Taken together, these findings suggest that the effects of erucic acid on plant growth and development depend on species context and treatment concentration.

### 3.5 Protein complex formation is essential for understanding the mechanism of erucic acid synthesis

Previous studies have shown that MED25 functions as a coactivator of MYC2 in regulating hormone-mediated immune responses in tomato (Liu et al., 2019). MED25 also plays a vital role in organ and tissue development. For instance, MED25 functions as a coactivator of PIF4 in regulating shade-induced hypocotyl elongation in tomato (Sun et al., 2020) and as a coactivator of DST in controlling spikelet number in rice (Lin et al., 2022). Our results also indicate that BnaMED25 physically interacts with BnaABI5 in the modulation of erucic acid biosynthesis (**Figures 3**, **4**). In addition, MED25 itself is involved in the regulation of plant growth and development. For example, *MED25* knockdown mutants exhibit altered fruit ripening in tomato (Deng et al., 2023). To further elucidate the role of BnaMED25 in regulating erucic acid biosynthesis, it will be important to determine whether BnaMED25 can function independently of its interacting partners. This distinction is important for determining whether BnaMED25 functions solely as a recruited cofactor or also exerts partially independent regulatory roles, thereby improving our understanding of the complex regulatory network governing erucic acid biosynthesis. Meanwhile, protein–protein interactions are widely observed across multiple plant species. In tomato, MED25 and JAZ7 competitively interact with MYC2 (Liu et al., 2019). In maize seeds, the E3 ubiquitin ligase ZmRFWD3 interacts with the master transcription factor Opaque2, while *Zm*SnRK1α2 phosphorylates ZmRFWD3 at Ser479, thereby reducing its stability through protein interaction (Li et al., 2020). During floral meristem activity and flower development, AtADA2b, a cofactor of the acetyltransferase GCN5, interacts with the chromatin-remodeling factor SYD and cytokinin-responsive B-type transcription factors (ARRs). These SYD–ADA2b–ARR interactions have also been demonstrated *in vivo* and *in vitro* (Hawar et al., 2025). C-TERMINAL DOMAIN PHOSPHATASE-LIKE3 (CPL3), a plant RNA polymerase II C-terminal domain phosphatase, interacts with FLOWERING LOCUS C EXPRESSOR (FLX) and FLX-LIKE4 (FLX4). Moreover, CPL3 interacts with FLX4 via FLX as a bridging factor to regulate flowering (Shen et al., 2022). In *Arabidopsis* and bread wheat, JAZ proteins interact with ABI5 to regulate seed germination (Ju et al., 2019). Furthermore, AtMED25 negatively regulates ABA signaling by interacting with AtABI5 (Chen et al., 2012a). In this study, we verified that BnaMED25 negatively affects the transcription of erucic acid biosynthesis-related genes by interacting with BnaABI5, and we further found that BnaABI5 interacts with BnaJAZ1 through Y2H screening and BiFC assays (data not shown). Considering the widespread occurrence of protein–protein interactions in plant regulatory networks, we propose that a BnaMED25–BnaABI5–BnaJAZ1 module may play an important role in regulating erucic acid biosynthesis. Future work should focus on identifying additional proteins that interact with BnaABI5 and characterizing their roles in regulating fatty acid biosynthesis.

## 4 Conclusion

Expression profiling and comprehensive functional characterization of BnaABI5 were conducted in this study. *BnaABI5* exhibited differential expression across tissues, whereas *BnaFAE1* showed seed-specific high expression. BnaABI5 functions as a nuclear-localized transcription factor and activates reporter gene expression in a yeast system. BnaABI5 positively regulates *BnaFAE1* expression by directly binding to ABRE cis-elements in its promoter. BnaABI5 interacts with BnaMED25, as demonstrated by multiple *in vitro* and *in vivo* assays. Furthermore, BnaMED25 represses BnaABI5-mediated activation of *BnaFAE1* expression. These findings broaden our understanding of the role of ABI5 in mediating very-long-chain fatty acid biosynthesis in *B. napus*.

## 5 Materials and methods

### 5.1 Plant materials and growth conditions

*A. thaliana* ecotype Col-0 was used as the wild-type control, and *med25* gene T-DNA insertional mutant *pft1–1* was previously described (Cerdán and Chory, 2003). Unless otherwise stated, all mutants used in this study are in the Columbia background. Genetic transformation was performed using the floral-dip method mediated by *Agrobacterium tumefaciens*. Transgenic *Arabidopsis* lines (*35S_pro_: BnaABI5-GFP*, *BnaABI5_pro_:GUS*, and *BnaFAE1_pro_:GUS*) and the mutant *abi5–7* were identified by PCR, antibiotic selection, and/or sequencing. Seeds of wild-type, transgenic, and mutant *Arabidopsis* plants were surface-sterilized with 70% (v/v) ethanol and washed three times with sterile water. The seeds were then sown in pots filled with a mixture of nutrient soil and vermiculite (2:1, v/v). Seedlings were grown in a growth chamber at 22 °C under a diurnal cycle of 16 h light and 8 h dark. The oilseed rape (*B. napus*) cultivar “Wangxiangyou1881” and transgenic *35S_pro_: BnaABI5-GFP* lines were grown under field conditions with appropriate isolation and management practices.

### 5.2 Plasmid construction

The cloning vector pEASY-T1 was purchased from TransGen Co., Ltd. (Beijing, China). To amplify the CDSs of *BnaABI5* and *BnaMED25*, RT-PCR was performed using primers listed in **Supplementary Table 1**. The amplified fragments were ligated into the pEASY-T1 vector to generate *pEASY-BnaABI5* and *pEASY-BnaMED25*, respectively. To generate constructs for GUS histochemical analysis, the promoter sequences of *BnaABI5* and *BnaFAE1* were amplified from genomic DNA of “Wangxiangyou1881” and cloned into the pCAMBIA1300-GUS vector to produce *BnaABI5_pro_:GUS* and *BnaFAE1_pro_:GUS*, respectively. For subcellular localization analysis, the full-length CDS of *BnaABI5* was cloned into pCAMBIA1302-GFP to generate the *35S_pro_: BnaABI5-GFP* construct. To assess potential self-activation, the full-length CDS of *BnaABI5* was cloned into pGBKT7 to generate *pGBKT7-BnaABI5*. For Y2H assays, the full-length CDS and truncated fragments of BnaABI5 were cloned into pGADT7, while the full-length CDS and truncated fragments of BnaMED25 were cloned into pGBKT7, generating *pGADT7-BnaABI5*, *pGBKT7-BnaMED25*, and their corresponding truncated constructs. For BiFC assays, *BnaABI5* was cloned into pXY104 and *BnaMED25* into pXY106, generating *pXY104-BnaABI5* and *pXY106-BnaMED25*, respectively. For pull-down assays, the full-length CDSs of *BnaMED25* and *BnaABI5* were cloned into pET-28a(+) and 18T vectors, respectively. For Co-IP assays, the full-length CDSs of *BnaABI5* and *BnaMED25* were cloned into pBWA(V)Hs-GFP and pBWA(V)Hs-Myc, respectively. For functional analysis, the construct used was identical to that used for subcellular localization. For Y1H assays, fragments of the *BnaFAE1* promoter, including three truncated fragments, were cloned into pABAi to generate *BnaFAE1pro-pABAi* and its truncated derivatives (*BnaFAE1pro-1-pABAi*, *BnaFAE1pro-2-pABAi*, and *BnaFAE1pro-3-pABAi*). For dual-luciferase reporter assays, *BnaABI5* and *BnaMED25* were cloned into pGreenII 62-SK as effector constructs. The second truncated fragment of the *BnaFAE1* promoter was cloned into pGreenII 0800-LUC as the reporter construct. All primers used for vector construction are listed in **Supplementary Table 1**.

### 5.3 Expression profile analysis of *BnaABI5* and *BnaFAE1*

To assess whether BnaABI5 is involved in regulating erucic acid accumulation, the expression profiles of *BnaABI5* and *BnaFAE1* were analyzed using the BnIR database (https://yanglab.hzau.edu.cn/BnIR). Using the “Expression Profile (ZS11 library)” module of the transcriptomics portal, *BnaABI5* and *BnaFAE1* were queried by entering their gene IDs, and “tissue” was selected as the column parameter; output data, including phylogenetic relationships, heatmaps, and expression charts, were retrieved from the gene information interface. Spatiotemporal expression patterns of *BnaABI5* and *BnaMED25* were compared and analyzed using the same database.

### 5.4 Quantitative real-time PCR analysis

The Super FastPure Cell RNA Isolation Kit was obtained from Vazyme Co., Ltd. (Nanjing, Jiangsu, China), and the Advantage RT-for-PCR Kit was purchased from Takara Bio Inc. (Dalian, Liaoning, China). Total RNA was isolated from rosette leaves of 4-week-old plants of different genetic backgrounds using the Super FastPure Cell RNA Isolation Kit (Cat. No. RC102-01), and reverse transcription was performed using the Advantage RT-for-PCR Kit (Cat. No. 639505). The *Arabidopsis ACTIN2* gene was used as the internal reference gene. The gene-specific primers used for qRT-PCR are listed in **Supplementary Table 1**. qRT-PCR analyses were performed using the Bio-Rad CFX96 Real-Time PCR System. Relative expression levels were calculated using the 2^−ΔΔCT^ method as previously described (Livak and Schmittgen, 2001).

### 5.5 GUS histochemical staining

For GUS staining, tissues including seedlings, leaves with petioles, inflorescences, siliques, and seeds from *BnaABI5_pro_:GUS* and *BnaFAE1_pro_:GUS* transgenic *A. thaliana* plants were collected and immersed in GUS staining solution containing 0.1 M phosphate buffer, 1% (v/v) Triton X-100, 0.25 mM Na_2_EDTA, 25 mM K_3_[Fe(CN)_6_], and 25 mM K_4_[Fe(CN)_6_]·3H_2_O. After incubation for 1 h at 37°C, the samples were cleared in an ethanol series (70% and 100%) to remove the chlorophyll. GUS staining signals in the treated tissues were observed and imaged using a stereomicroscope (Olympus).

### 5.6 Subcellular localization assay

A C-terminal GFP fusion of *BnaABI5* was generated as described in Section 5.2. The recombinant vector and the empty pCAMBIA1302 vector were introduced into *A. tumefaciens* strain GV3101 and cultured overnight at 28 °C with shaking. An aliquot (1 mL) of the overnight culture was used to inoculate 50 mL of Luria–Bertani (LB) medium supplemented with appropriate antibiotics, 10 mM MES, and 20 µM acetosyringone. After centrifugation at 5,000 rpm for 10 min at 4 °C, the cell pellet was resuspended in infiltration buffer (10 mM MgCl_2_, 10 mM MES, and 100 µM acetosyringone) to a final OD_600_ of 1.0. The bacterial suspensions were then infiltrated into leaves of approximately 4-week-old *N. benthamiana* plants. After 2 days, infiltrated leaves were collected, and fluorescence signals were observed using a Nikon A1 confocal laser scanning microscope.

### 5.7 Transcriptional activation assay

The recombinant plasmid *pGBKT7-BnaABI5* and the corresponding empty vector pGBKT7 were transformed into the yeast strain AH109. Positive yeast transformants were selected and cultured on synthetic dropout (SD) medium lacking tryptophan (SD/−Trp) for 2–3 days. To assess transcriptional activation activity, the transformants were further grown on SD medium lacking histidine (SD/−His) supplemented with X-α-gal to evaluate reporter gene activation based on color development.

### 5.8 Genetic transformation and identification

The corresponding vectors were constructed as described in Section 5.2. Full-length CDSs or promoter sequences were overexpressed in *A. thaliana* using the floral-dip method (Clough and Bent, 1998) mediated by *A. tumefaciens* strain GV3101. Seeds from transgenic plants were harvested, and lines exhibiting a Mendelian segregation ratio of 3:1 were selected and subsequently selfed to obtain homozygous lines. For validation, gene expression levels were quantified by qRT-PCR. Transgenic *B. napus* plants were generated via *A. tumefaciens*-mediated transformation of hypocotyl explants as previously described (Dai et al., 2020; Zhang et al., 2025c), and gene expression levels in overexpression lines were also analyzed by qRT-PCR.

### 5.9 Yeast two-hybrid assay

To verify the interaction between BnaABI5 and BnaMED25, full-length CDSs and corresponding truncated fragments were co-transformed into AH109 yeast-competent cells. Co-transformants were initially grown on SD medium lacking leucine and tryptophan (SD/−Leu/−Trp). To eliminate potential self-activation of bait proteins, colonies were transferred to SD medium lacking adenine, histidine, leucine, and tryptophan (SD/−Ade/−His/−Leu/−Trp) supplemented with an appropriate concentration of 3-amino-1,2,4-triazole (3-AT) and incubated at 30 °C for 3–4 days. α-Galactosidase activity was assessed by monitoring *MEL1* reporter gene expression on selection plates supplemented with X-α-gal. The SV40 large T antigen fused to pGADT7 and p53 fused to pGBKT7 were used as positive controls.

### 5.10 Bimolecular fluorescence complementation assays

Vector construction is described in Section 5.2. After sequence verification, the constructs were introduced into *A. tumefaciens* strain GV3101 and transiently expressed in 5-week-old *N. benthamiana* leaves via agroinfiltration. Following infiltration, plants were maintained under controlled growth conditions at approximately 22–25 °C for 48 h before observation. YFP fluorescence signals were observed using a confocal laser scanning microscope (Zeiss LSM-710).

### 5.11 Cell-free protein expression

Vector construction for plasmid generation is described in Section 5.2. BnaABI5-FLAG and BnaMED25-His proteins were synthesized using the ProteinFactory cell-free expression kit according to the manufacturer’s instructions. The procedure was performed as follows: Amplified recombinant plasmid templates were added to the ProteinFactory reaction system at the recommended proportions and adjusted with water to a final volume of 10 mL. The reaction mixture was incubated at approximately 22–25 °C with shaking at 200 rpm for 3 h to allow protein synthesis. Protein expression was carried out using a wheat germ cell-free system as previously described (Harbers, 2014). The expressed proteins were detected using specific antibodies. Confirmed protein products were collected for subsequent experiments.

### 5.12 Co-immunoprecipitation assays

Vector construction is described in Section 5.2. To examine the interaction between BnaABI5 and BnaMED25 *in vivo*, BnaMED25-GFP and Myc-BnaABI5 were transiently co-expressed in *N. benthamiana* leaves, and protein expression was confirmed by immunoblotting. For immunoprecipitation, GFP-Trap or Myc-Trap agarose beads (ChromoTek) were used. Immunoblots were probed with anti-Myc and anti-GFP antibodies (Abcam).

### 5.13 Pull-down assay

Vectors used for the experiment were generated as described in Section 5.2. Recombinant protein expression was performed using a cell-free expression system. For pull-down assays, His-tag affinity magnetic beads were incubated with protein mixtures from each group at 4 °C for 4 h, followed by three washes with phosphate-buffered saline (PBS). After elution from the beads, bound proteins were separated by SDS-PAGE. His-tagged proteins, including BnaMED25-His, were detected using an anti-His antibody (1:4,000 dilution; Nanjing Zoonbio Biotechnology Co., Ltd., China; catalog no. 10010), whereas BnaABI5-FLAG was detected using an anti-FLAG antibody (1:5,000 dilution; Nanjing Zoonbio Biotechnology Co., Ltd., China; catalog no. F1804).

### 5.14 Bioinformatics analysis

Putative cis-acting elements in the *BnaFAE1* promoter were predicted using the PlantCARE database (http://bioinformatics.psb.ugent.be/webtools/plantcare/html/). Promoter sequences were submitted to PlantCARE for analysis of regulatory motifs.

### 5.15 Yeast one-hybrid assay

The intact *BnaFAE1* promoter was divided into three truncated fragments based on bioinformatic analysis. Recombinant constructs containing the intact *BnaFAE1* promoter and its truncated fragments were linearized and transformed into Y1H Gold competent cells. Positive clones were confirmed by PCR and used to determine the optimal AbA concentration required to suppress background activation. The *pGADT7-BnaABI5* construct was transformed into bait strains harboring *pAbAi* constructs containing the *BnaFAE1* promoter or its truncated fragments. Transformed yeast clones were cultured on SD/−Leu medium supplemented with varying concentrations of AbA at 30 °C for 3–4 days. *pGADT7-p53* co-transformed with *pAbAi-p53* was used as a positive control, whereas *pGADT7* empty vector co-transformed with *pAbAi-p53* served as a negative control.

### 5.16 Dual-luciferase reporter assay

The second fragment of the *BnaFAE1* promoter was inserted into the pGreenII 0800-LUC vector as the reporter construct, whereas BnaABI5 and BnaMED25 were cloned into the pGreenII 62-SK vector as effector constructs; *35Spro: REN* served as an internal control, and the empty vector was used as a negative control. After sequence verification, the effector and reporter constructs were introduced into *A. tumefaciens* strain GV3101. *Agrobacterium* cultures were resuspended in infiltration buffer as previously described (Xue et al., 2023). The bacterial suspensions were infiltrated into the leaves of 2-week-old *N. benthamiana* plants. LUC and REN activities were quantified 48 h after infiltration using a dual-luciferase assay system (Promega, USA) and a chemiluminescence imaging system (Fusion FX7, Vilber).

### 5.17 Electrophoretic mobility shift assay

The vectors used in this assay were the same as those described for the pull-down assay. A DNA probe containing the ABRE element from the *BnaFAE1* promoter was synthesized and biotin-labeled at the 3′ end (Tsingke Biotechnology, China). The biotin-labeled DNA probes were incubated with BnaABI5-FLAG protein in binding buffer. For competition assays, an unlabeled probe was added at 50× and 100× molar excess relative to the labeled probe. Mutated probes were included at 1× and 50× molar excess relative to the labeled probe. After separation on 6% non-denaturing polyacrylamide gels (PAGE), protein–DNA complexes and free probes were transferred to a nitrocellulose membrane. Signals were detected using a chemiluminescent detection kit (Thermo Fisher Scientific, USA).

### 5.18 Gas chromatography analysis of erucic acid

Erucic acid content in *A. thaliana* seeds was determined by gas chromatography (GC) as previously described (Chen et al., 2012b). Briefly, intact seeds (~10 mg) were subjected to acid-catalyzed methanolysis using 1 M HCl in methanol at 80 °C for 2 h. The resulting fatty acid methyl esters (FAMEs) were extracted into hexane following the addition of an aqueous NaCl solution (0.9% w/v). The hexane phase was analyzed using a Shimadzu GC-2014 gas chromatograph equipped with a flame ionization detector (FID) and a Supelco Wax-10 capillary column (30 m × 0.25 mm × 0.5 μm). Chromatographic separation was achieved using an oven program consisting of a 1-min hold at 160 °C, followed by a temperature ramp of 4 °C min^−1^ to 240 °C, and a final 16-min isothermal hold. Peaks were identified by comparison of retention times, and quantification was performed by normalizing peak areas to that of the methyl heptadecanoate internal standard.

### 5.19 Near-infrared spectroscopy analysis of erucic acid

Erucic acid content in oilseed rape seeds was determined using a Foss near-infrared spectrometer (Büchi NIR Transport System B291) according to the manufacturer’s protocol, with measurements processed using OPUS software and instrument-specific calibration models. Briefly, approximately 6 g of rapeseed seeds per sample were placed in a sample holder attached to the instrument. Spectral data were acquired and analyzed automatically, and results were displayed on the computer interface.

### 5.20 Statistical analysis

Statistical analyses were performed using Student’s *t*-test or one-way analysis of variance (ANOVA) in GraphPad Prism (version 10.0). A *p*-value < 0.05 was considered statistically significant; differences among groups were indicated by different letters.

## Data Availability

The original contributions presented in the study are included in the article/**Supplementary Material**, further inquiries can be directed to the corresponding author/s.

## Supplementary material

Supplementary Figure 1 Spatiotemporal expression patterns of multiple *BnaABI5* gene copies. **(A)** Spatiotemporal expression profiles of *BnaABI5* genes are shown as a heatmap. Tissues include bud, filament, pollen, cotyledon, leaf, seed, and silique at different days after fertilization, as well as stem tissues from different positions. Expression levels are represented by different colors. Yellow and green indicate high and low expression levels, respectively. **(B)** Expression values of *BnaABI5* genes are presented as line charts. The tissues analyzed are primarily the same as those shown in **(A)**. **(C)** Expression values of the *BnaABI5* genes are presented as bar charts. Tissues analyzed include bud, flower, leaf, root, seed, silique, and stem.

Supplementary Figure 2 Spatiotemporal expression patterns of multiple *BnaFAD2* gene copies. **(A)** Spatiotemporal expression profiles of *BnaFAD2* genes shown as a heatmap. Tissues include bud, filament, pollen, cotyledon, leaf, seed, and silique at different days after fertilization, as well as stem tissues from different positions. Expression levels are represented by different colors. Yellow and green indicate high and low expression levels, respectively. **(B)** Expression values of *BnaFAD2* genes are presented as line charts. The tissues analyzed are primarily the same as those shown in **(A)**. **(C)** Expression values of *BnaFAD2* genes are presented as bar charts. Tissues analyzed include bud, flower, leaf, root, seed, silique, and stem.

Supplementary Figure 3 Spatiotemporal expression patterns of multiple *BnaFAD3* gene copies. **(A)** Spatiotemporal expression profiles of *BnaFAD3* genes shown as a heatmap. Tissues include bud, filament, pollen, cotyledon, leaf, seed, and silique at different days after fertilization, as well as stem tissues from different positions. Expression levels are represented by different colors. Yellow and green indicate high and low expression levels, respectively. **(B)** Expression values of *BnaFAD3* genes are presented as line charts. The tissues analyzed are primarily the same as those shown in **(A)**. **(C)** Expression values of *BnaFAD3* genes are presented as bar charts. Tissues analyzed include bud, flower, leaf, root, seed, silique, and stem.

Supplementary Figure 4 Spatiotemporal expression patterns of multiple *BnaFAE1* gene copies. **(A)** Spatiotemporal expression profiles of *BnaFAE1* genes shown as a heatmap. Tissues include bud, filament, pollen, cotyledon, leaf, seed, and silique at different days after fertilization, as well as stem tissues from different positions. Expression levels are represented by different colors. Yellow and green indicate high and low expression levels, respectively. **(B)** Expression values of *BnaFAE1* genes are presented as line charts. The tissues analyzed are primarily the same as those shown in **(A)**. **(C)** Expression values of *BnaFAE1* genes are presented as bar charts. Tissues analyzed include bud, flower, leaf, root, seed, silique, and stem.

Supplementary Figure 5 Expression levels of the *GUS* reporter gene in *BnaABI5::GUS* transgenic *Arabidopsis*. **(A)** Relative expression levels of the *GUS* reporter gene in wild-type (Col-0) and transgenic *Arabidopsis* lines. *Actin2* was used as the internal reference gene. **(B)** Relative expression levels of the *GUS* reporter gene in different transgenic *Arabidopsis* lines. Asterisks indicate statistically significant differences. Error bars represent the mean ± standard deviation (SD) of three biological replicates.

Supplementary Figure 6 Expression levels of the *GUS* reporter gene in *BnaFAE1::GUS* transgenic *Arabidopsis*. **(A)** Expression of the *GUS* reporter gene was assessed by semi-quantitative PCR in wild-type (Col-0) and transgenic *Arabidopsis* lines. *Actin2* was used as the internal reference gene. **(B)** Relative expression levels of the *GUS* reporter gene were quantified by qRT-PCR in different transgenic *Arabidopsis* lines. Asterisks indicate statistically significant differences. Error bars represent the mean ± standard deviation (SD) of three biological replicates.

Supplementary Figure 7 Expression levels of the *BnaABI5* gene in *35S::BnaABI5* transgenic *Arabidopsis*. **(A)** Expression of the *BnaABI5* gene was assessed by semi-quantitative PCR in wild-type (Col-0) and transgenic *Arabidopsis* lines. *Actin2* was used as the internal reference gene. **(B)** Relative expression levels of the *BnaABI5* gene were quantified by qRT-PCR in different transgenic *Arabidopsis* lines. Asterisks indicate statistically significant differences. Error bars represent the mean ± standard deviation (SD) of three biological replicates.

Supplementary Figure 8 Comparative sequence alignment of a defined genomic region of the *AtABI5* locus between wild-type *Arabidopsis thaliana* (Col-0 ecotype) and the *abi5–7* mutant. The region was amplified from Col-0 ecotype (top row) and the *abi5–7* mutant background (bottom row) using the same primers, and the target fragment was subsequently sequenced. All experiments yielded similar results across at least three biological replicates, including independent sequencing analyses.

Supplementary Figure 9 Expression levels of *BnaABI5* gene in *35S::BnaABI5* transgenic oilseed rape. Relative expression levels of the *BnaABI5* gene were quantified by qRT-PCR in different transgenic oilseed rape lines. *Actin7* was used as the internal reference gene. Asterisks indicate statistically significant differences. Error bars represent the mean ± standard deviation (SD) of three biological replicates.

Supplementary Figure 10 Expression level of *AtFAE1* gene in in wild-type Col-0, mutant *abi5–7* and *pft1-1*. *Actin2* gene was served as internal reference. Asterisk indicates statistically significant difference. Error bars represent the mean ± standard deviation (SD) of three biological replicates.
