# Supplementary figures and images for: Mediator subunit MED25 represses ABI5-dependent activation of erucic acid biosynthetic gene FAE1 in Brassica napus
Source: Front Plant Sci. 2026 Jun 10;17:1798651. doi: 10.3389/fpls.2026.1798651 (PMC13290517; doi:10.3389/fpls.2026.1798651)

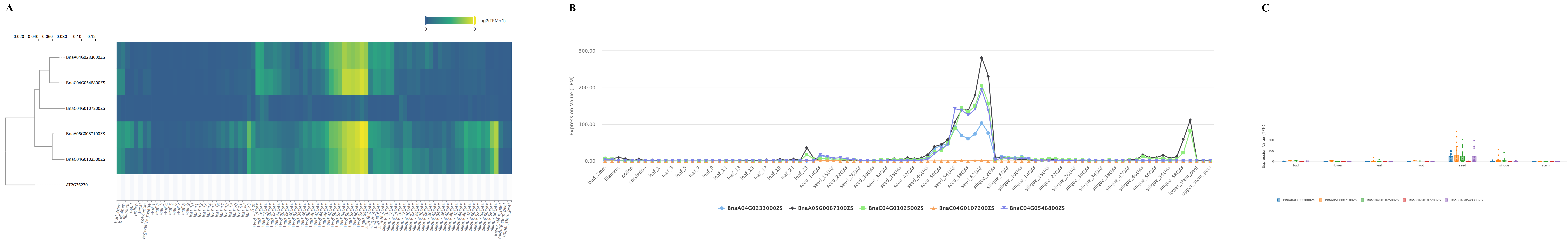

Supplement: Supplementary Figure 1 — Spatiotemporal expression patterns of multiple BnaABI5 gene copies. (A) Spatiotemporal expression profiles of BnaABI5 genes are shown as a heatmap. Tissues include bud, filament, pollen, cotyledon, leaf, seed, and silique at different days after fertilization, as well as stem tissues from different positions. Expression levels are represented by different colors. Yellow and green indicate high and low expression levels, respectively. (B) Expression values of BnaABI5 genes are presented as line charts. The tissues analyzed are primarily the same as those shown in (A). (C) Expression values of the BnaABI5 genes are presented as bar charts. Tissues analyzed include bud, flower, leaf, root, seed, silique, and stem. [file Image1.tif]

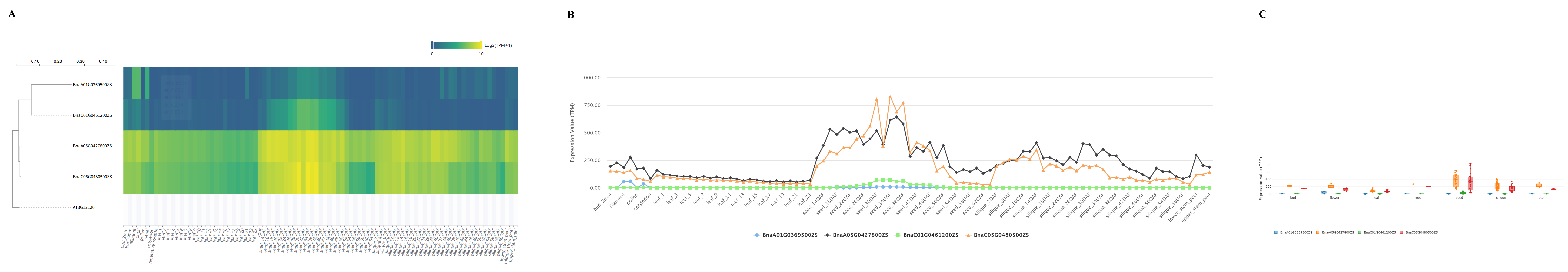

Supplement: Supplementary Figure 2 — Spatiotemporal expression patterns of multiple BnaFAD2 gene copies. (A) Spatiotemporal expression profiles of BnaFAD2 genes shown as a heatmap. Tissues include bud, filament, pollen, cotyledon, leaf, seed, and silique at different days after fertilization, as well as stem tissues from different positions. Expression levels are represented by different colors. Yellow and green indicate high and low expression levels, respectively. (B) Expression values of BnaFAD2 genes are presented as line charts. The tissues analyzed are primarily the same as those shown in (A). (C) Expression values of BnaFAD2 genes are presented as bar charts. Tissues analyzed include bud, flower, leaf, root, seed, silique, and stem. [file Image2.tif]

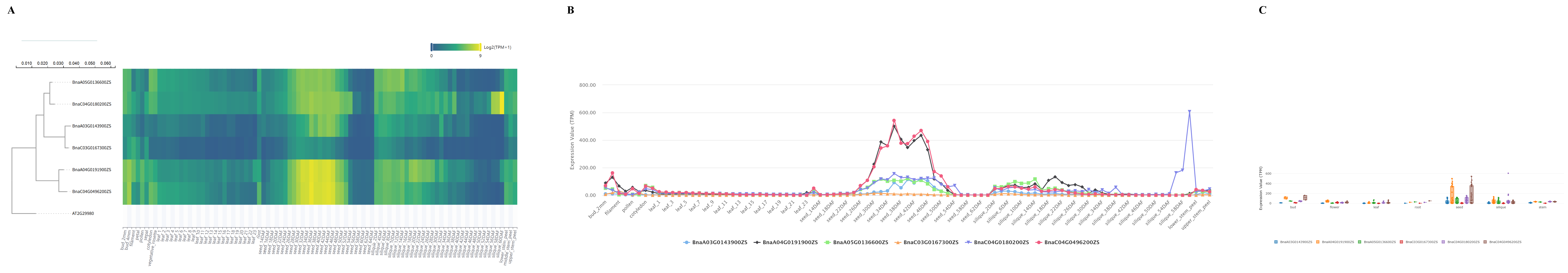

Supplement: Supplementary Figure 3 — Spatiotemporal expression patterns of multiple BnaFAD3 gene copies. (A) Spatiotemporal expression profiles of BnaFAD3 genes shown as a heatmap. Tissues include bud, filament, pollen, cotyledon, leaf, seed, and silique at different days after fertilization, as well as stem tissues from different positions. Expression levels are represented by different colors. Yellow and green indicate high and low expression levels, respectively. (B) Expression values of BnaFAD3 genes are presented as line charts. The tissues analyzed are primarily the same as those shown in (A). (C) Expression values of BnaFAD3 genes are presented as bar charts. Tissues analyzed include bud, flower, leaf, root, seed, silique, and stem. [file Image3.tif]

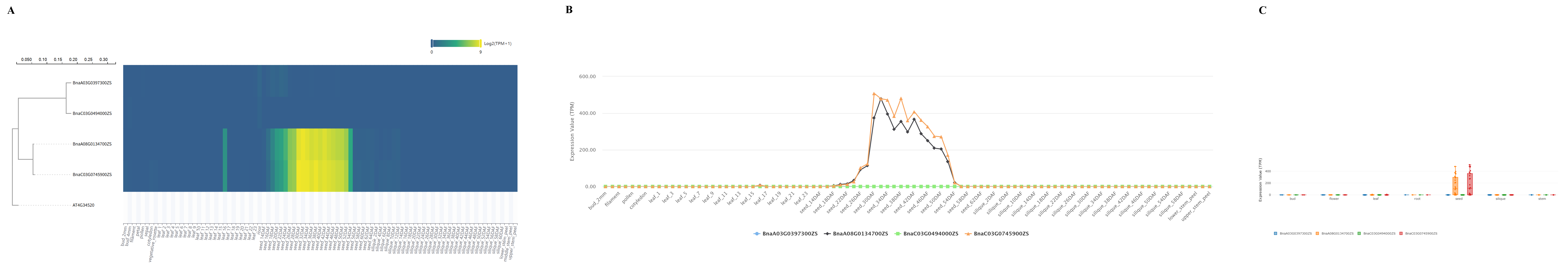

Supplement: Supplementary Figure 4 — Spatiotemporal expression patterns of multiple BnaFAE1 gene copies. (A) Spatiotemporal expression profiles of BnaFAE1 genes shown as a heatmap. Tissues include bud, filament, pollen, cotyledon, leaf, seed, and silique at different days after fertilization, as well as stem tissues from different positions. Expression levels are represented by different colors. Yellow and green indicate high and low expression levels, respectively. (B) Expression values of BnaFAE1 genes are presented as line charts. The tissues analyzed are primarily the same as those shown in (A). (C) Expression values of BnaFAE1 genes are presented as bar charts. Tissues analyzed include bud, flower, leaf, root, seed, silique, and stem. [file Image4.tif]

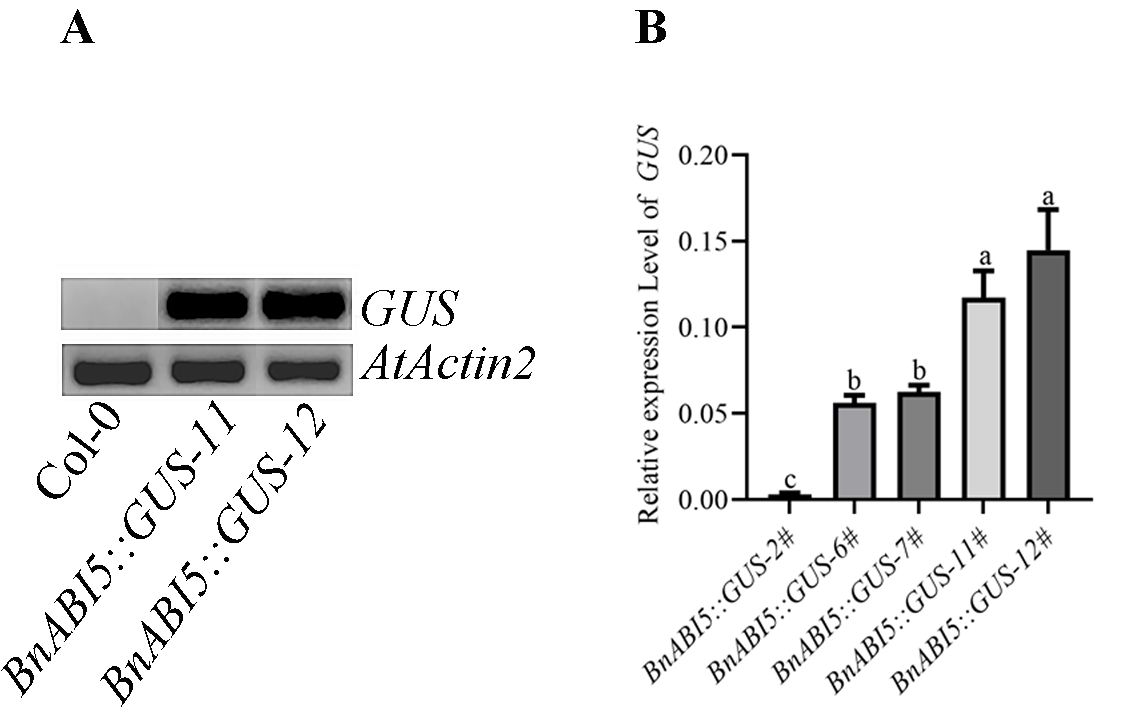

Supplement: Supplementary Figure 5 — Expression levels of the GUS reporter gene in BnaABI5::GUS transgenic Arabidopsis. (A) Relative expression levels of the GUS reporter gene in wild-type (Col-0) and transgenic Arabidopsis lines. Actin2 was used as the internal reference gene. (B) Relative expression levels of the GUS reporter gene in different transgenic Arabidopsis lines. Asterisks indicate statistically significant differences. Error bars represent the mean ± standard deviation (SD) of three biological replicates. [file Image5.tif]

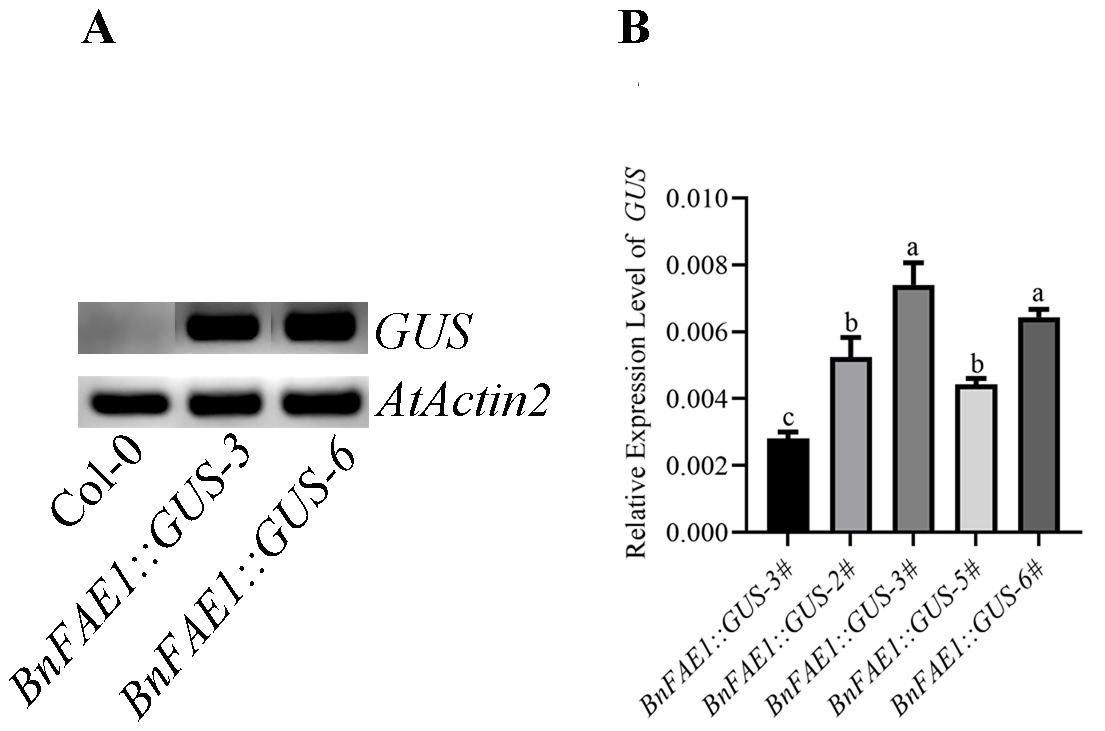

Supplement: Supplementary Figure 6 — Expression levels of the GUS reporter gene in BnaFAE1::GUS transgenic Arabidopsis. (A) Expression of the GUS reporter gene was assessed by semi-quantitative PCR in wild-type (Col-0) and transgenic Arabidopsis lines. Actin2 was used as the internal reference gene. (B) Relative expression levels of the GUS reporter gene were quantified by qRT-PCR in different transgenic Arabidopsis lines. Asterisks indicate statistically significant differences. Error bars represent the mean ± standard deviation (SD) of three biological replicates. [file Image6.tif]

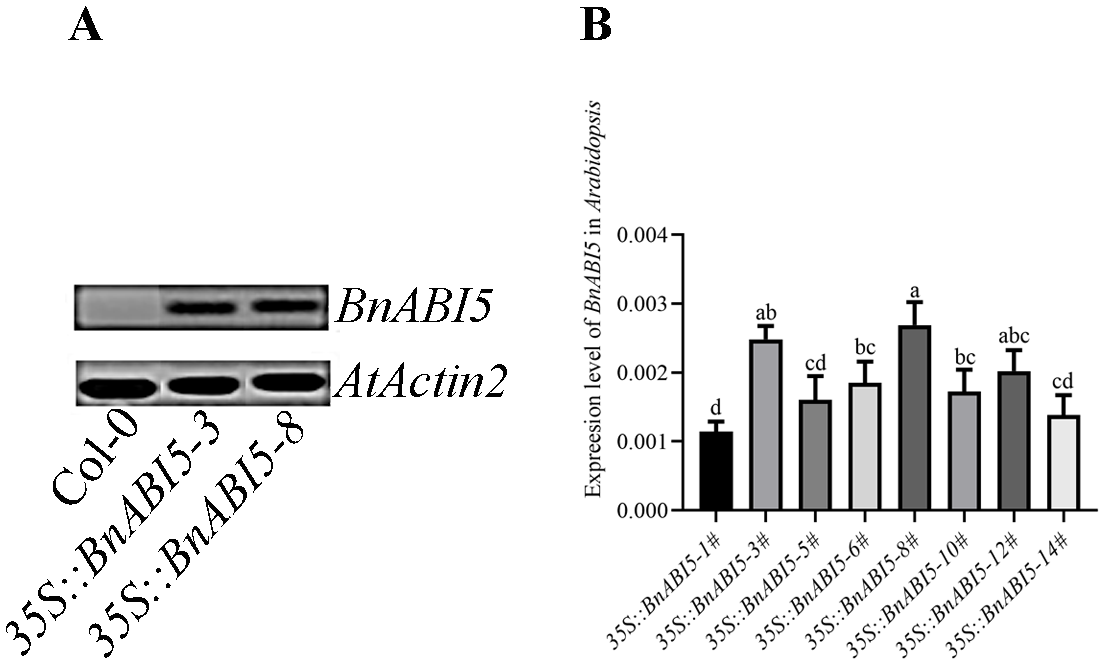

Supplement: Supplementary Figure 7 — Expression levels of the BnaABI5 gene in 35S::BnaABI5 transgenic Arabidopsis. (A) Expression of the BnaABI5 gene was assessed by semi-quantitative PCR in wild-type (Col-0) and transgenic Arabidopsis lines. Actin2 was used as the internal reference gene. (B) Relative expression levels of the BnaABI5 gene were quantified by qRT-PCR in different transgenic Arabidopsis lines. Asterisks indicate statistically significant differences. Error bars represent the mean ± standard deviation (SD) of three biological replicates. [file Image7.tif]

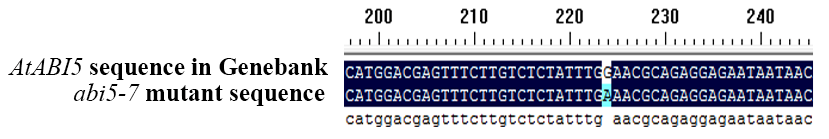

Supplement: Supplementary Figure 8 — Comparative sequence alignment of a defined genomic region of the AtABI5 locus between wild-type Arabidopsis thaliana (Col-0 ecotype) and the abi5–7 mutant. The region was amplified from Col-0 ecotype (top row) and the abi5–7 mutant background (bottom row) using the same primers, and the target fragment was subsequently sequenced. All experiments yielded similar results across at least three biological replicates, including independent sequencing analyses. [file Image8.tif]

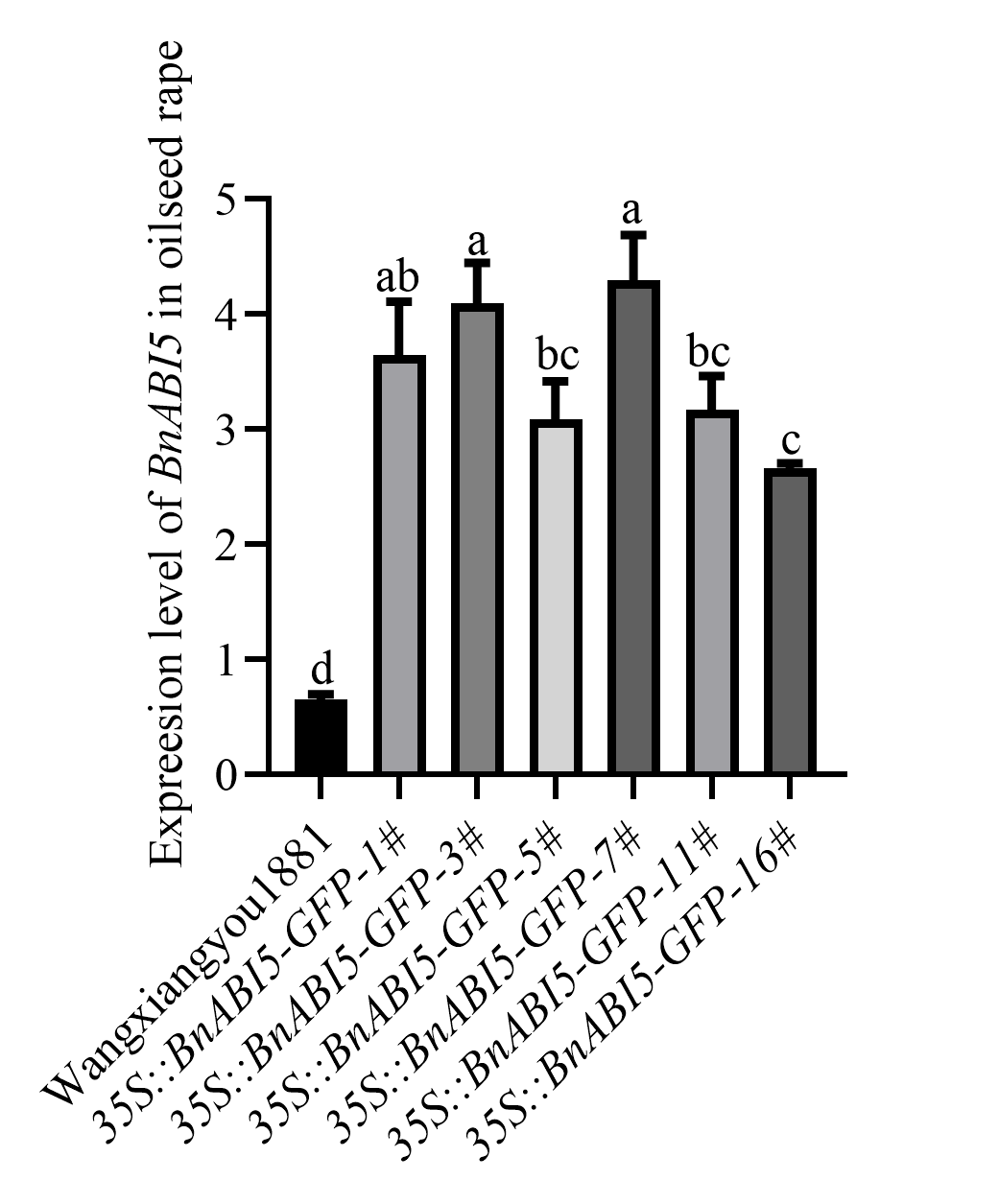

Supplement: Supplementary Figure 9 — Expression levels of BnaABI5 gene in 35S::BnaABI5 transgenic oilseed rape. Relative expression levels of the BnaABI5 gene were quantified by qRT-PCR in different transgenic oilseed rape lines. Actin7 was used as the internal reference gene. Asterisks indicate statistically significant differences. Error bars represent the mean ± standard deviation (SD) of three biological replicates. [file Image9.tif]

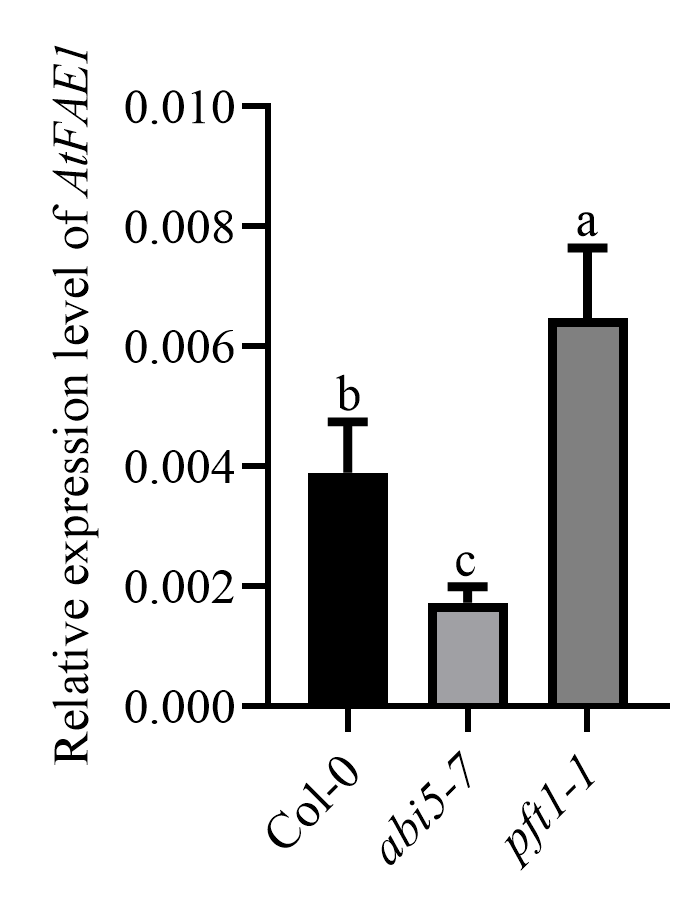

Supplement: Supplementary Figure 10 — Expression level of AtFAE1 gene in in wild-type Col-0, mutant abi5–7 and pft1-1. Actin2 gene was served as internal reference. Asterisk indicates statistically significant difference. Error bars represent the mean ± standard deviation (SD) of three biological replicates. [file Image10.tif]
