# Supplementary material for: Mediator subunit MED25 represses ABI5-dependent activation of erucic acid biosynthetic gene FAE1 in Brassica napus
Source: Front Plant Sci. 2026 Jun 10;17:1798651. doi: 10.3389/fpls.2026.1798651 (PMC13290517; doi:10.3389/fpls.2026.1798651)
Supplement: Supplementary file 11 [file Table1.docx]

Supplemental Table 1. DNA primers and probes used in this study

| Primers for gene cloning | |
| --- | --- |
| BnaABI5-F | CATGGTGAGCAGAGAAACAGAG |
| BnaABI5-R | CGTCATCAGAGAGGGCAAC |
| BnaMED25-F | CAGCATGTCGTCGGAGTTG |
| BnaMED25-R | TTATCCCATGAAGCCCGCTC |
| Primers for subcellular localization and genetic transformation | |
| 1302-BnaABI5-F | ACTCTTGACCATGGTAGATCTATGGTGAGCAGAGAAACAGAG |
| 1302-BnaABI5-R | AAGTTCTTCTCCTTTACTAGTTCAGAGAGGGCAAC |
| Primers for GUS constructs | |
| 1301-BnaABI5-F | GACCTGCAGGCATGCAAGCTTATAATTCATGTGCGCTCGCAAGC |
| 1301-BnaABI5-R | TTACCCTCAGATCTACCATGGCTACCAGCTGCAGACAAAC |
| 1301-BnaFAE1-F | GAGCTCGGTACCCGGGGATCCTGTAAATAGTTGGGAAGTTATCTCC |
| 1301-BnaFAE1-R | TTACCCTCAGATCTACCATGGAGAGGAAGCGATGAGATG |
| Primers for Y2H | |
| AD- BnaABI5-F | GTACCAGATTACGCTCATATGATGGTGAGCAGAGAAACAGAG |
| AD- BnaABI5-R | CAGCTCGAGCTCGATGGATCCTCATCAGAGAGGGCAAC |
| BD-BnaMED25-F | ATGGCCATGGAGGCCGAATTCATGTCGTCGGAGTTG |
| BD-BnaMED25-R | CCGCTGCAGGTCGACGGATCCTTATCCCATGAAG |
| AD-BnaABI5-C1-F | GTACCAGATTACGCTCATATGATGATGTCTGAACGAGAAG |
| AD-BnaABI5-C1-R | CAGCTCGAGCTCGATGGATCCTCAGGAGCCACCACCACC |
| AD-BnaABI5-C2-F | GTACCAGATTACGCTCATATGATGGCCGCAGCTTCACATC |
| AD-BnaABI5-C2-R | CAGCTCGAGCTCGATGGATCCTCAGTTATTAGCATTGTT |
| AD-BnaABI5-C3-F | GTACCAGATTACGCTCATATGATGGGGGATGGAGACAAC |
| AD-BnaABI5-C3-R | CAGCTCGAGCTCGATGGATCCTCAACCGCCGTAACAAAC |
| AD-BnaABI5-  bZIP-F | GTACCAGATTACGCTCATATGATGTTCCCGGTGGGTGTAG |
| AD-BnaABI5-  bZIP-R | CAGCTCGAGCTCGATGGATCCTCATTGCTTCCTCTTCCTC |
| AD-BnaABI5-  bZIP-C4-F | GTACCAGATTACGCTCATATGATGTTCCCGGTGGGTGTAG |
| AD-BnaABI5-  bZIP-C4-R | CAGCTCGAGCTCGATGGATCCTCAGAGAGGGCAACTAGG |
| BD-BnaMED25- vWF-A-F | ATGGCCATGGAGGCCGAATTCATGTCGTCGGAGTTG |
| BD-BnaMED25- vWF-A-R | CCGCTGCAGGTCGACGGATCCTCACTCTGAGATCAGGAC |
| BD-BnaMED25-MD-F | ATGGCCATGGAGGCCGAATTCATGAACTTTGTGGAGGCAC |
| BD-BnaMED25-MD-R | CCGCTGCAGGTCGACGGATCCTCACTGCATAGCCCC |
| BD-BnaMED25-ACID-F | ATGGCCATGGAGGCCGAATTCATGCCTTCTCAATCC |
| BD-BnaMED25-ACID-R | CCGCTGCAGGTCGACGGATCCTCAATTTTGAATTTGTGGC |
| BD-BnaMED25-GD-F | ATGGCCATGGAGGCCGAATTCATGCAGCAACAGCAGCAAC |
| BD-BnaMED25-GD-R | CCGCTGCAGGTCGACGGATCCTTATCCCATGAAG |
| Primers for BiFC | |
| BnaMED25-nYFP-F | ATCGAGGACGCCGGCGGATCCATGTCGTCGGAGTTG |
| BnaMED25-nYFP-R | GCTCTGCAGGTCGACTCTAGATTATCCCATGAAG |
| BnaABI5-cYFP-F | ATTACAGGTACCCGGGGATCCATGGTGAGCAGAGAAACAGAG |
| BnaABI5-cYFP-R | GCCACCGCCGTCGACTCAGAGAGGGCAACAAC |
| Primers for Pull-down and EMSA | |
| PD-BnaABI5-F | ACCACCATCACGGGAGCGGCAAGCTTATGGTGAGCAGAGAAACAGA |
| PD-BnaABI5-R | AAGTAATTAATCCTTATTTAGAATTCTCAGAGAGGGCAACTAGGGT |
| PD-BnaMED25-F | TATTCTCCCACAGTGGGAAAGGATCCATGTCGTCGGAGTTGAAACA |
| PD-BnaMED25-R | AAGTAATTAATCCTTATTTAGAATTCCTTGTCATCGTCGTCCTTGTA |
| Primers for Co-IP | |
| CoIP-BnaABI5-F | AAGAGGATTTGAATGGCTCCATGGTGAGCAGAGAAACAGAGATGATGTC |
| CoIP-BnaABI5-R | TCAGTCTTCGGGCCCCTAGTTCAGAGAGGGCAACTAGGGTTCC |
| CoIP-BnaMED25  -GFP-F | ACATTTACAATTACGGATCCATGTCGTCGGAGTTGAAACAGCTAATCG |
| CoIP-BnaMED25  -GFP-R | CCCTTGCTCACCATGGTACCTCCCATGAAGCCCGCTCCTG |
| Primers for Y1H | |
| pAbAi-BnaFAE1-P-F | CTTGAATTCGAGCTCGGTACCTGTAAATAGTTGGGAAG |
| pAbAi-BnaFAE1-P-R | ATACAGAGCACATGCCTCGAGAGAGGAAGCGATGAGATG |
| pAbAi-BnaFAE1-P1-F | CTTGAATTCGAGCTCGGTACCTGTAAATAGTTGGGAAG |
| pAbAi-BnaFAE1-P1-R | ATACAGAGCACATGCCTCGAGCGAAGACCGACTCCGCCT |
| pAbAi-BnaFAE1-P2-F | CTTGAATTCGAGCTCGGTACCGGTCTTCGGTTTCGGCCGAG |
| pAbAi-BnaFAE1-P2-R | ATACAGAGCACATGCCTCGAGCATCATAAACTCATTGC |
| pAbAi-BnaFAE1-P3-F | CTTGAATTCGAGCTCGGTACCGATCGGTAATAACCTTTC |
| pAbAi-BnaFAE1-P3-R | ATACAGAGCACATGCCTCGAGAGAGGAAGCGATGAGATG |
| Primers for dual-luciferase assay | |
| 62-SK-BnaABI5-F | CGCTCTAGAACTAGTGGATCCATGGTGAGCAGAGAAACAGAG |
| 62-SK-BnaABI5-R | GTCGACGGTATCGATAAGCTTTCATCAGAGAGGGCAAC |
| 62-SK-BnaMED25-F | CGCTCTAGAACTAGTGGATCCATGTCGTCGGAGTTG |
| 62-SK-BnaMED25-R | GTCGACGGTATCGATAAGCTTTTATCCCATGAAGCCCGCTC |
| BnaFAE1-P2-LUC-F | GTCGACGGTATCGATAAGCTTGGTCTTCGGTTTCGGCCG |
| BnaFAE1-P2-LUC-R | CGCTCTAGAACTAGTGGATCC CATCATAAACTCATTGCC |
| Probes for EMSA | |
| BnaFAE1-P2(SB) | TATGTGGTGACACGTGGTTTGAAAC |
| BnaFAE1-P2(M) | TATGTGGTGAAATTGGGTTTGAAAC |
| Primers for RT-PCR | |
| BnaABI5-F | CGAGTTCCAACACGCTCTCT |
| BnaABI5-R | GGCTGCTTAGCTATCCCTGG |
| GUS-F | TCTGGTATCAGCGCGAAGTC |
| GUS-R | CGGCAATAACATACGGCGTG |
| Primers for qRT-PCR | |
| BnaABI5-F | CAGCTTCACATCCCGTTCCA |
| BnaABI5-R | GGCTGCTTAGCTATCCCTGG |
| GUS-F | TCTGGTATCAGCGCGAAGTC |
| GUS-R | CGGCAATAACATACGGCGTG |
| AtFAE1-F | ATCGTAACCCGACCCAATCC |
| AtFAE1-R | TGCCACGTTCCGTGAAGAAG |
| BnaFAE1-F | AACCTCATAACCATCGCTCC |
| BnaFAE1-R | TCAAGAAGTCAAGCCACGAC |
| AtActin2-F | GGTAACATTGTGCTCAGTGGTGG |
| AtActin2-R | AACGACCTTAATCTTCATGCTGC |
| BnaActin7-F | TGCTCTTCCTCACGCTATCCTC |
| BnaActin7-R | GCTCGTAGTTCTTCTCCACCG |
| Primers for mutant identification | |
| abi5-7-F | TCAGAGCGAGAAGTAGAGTCG |
| abi5-7-R | CCACCACTAAAGACACCAACAC |
